# Identification of avoidance genes through neural pathway-specific forward optogenetics

**DOI:** 10.1371/journal.pgen.1008509

**Published:** 2019-12-31

**Authors:** Filipe Marques, Gabriella Saro, Andrei-Stefan Lia, Richard J. Poole, Laurent Falquet, Dominique A. Glauser

**Affiliations:** 1 Department of Biology, University of Fribourg, Fribourg, Switzerland; 2 Department of Cell and Developmental Biology, University College London, London, United Kingdom; 3 Swiss Institute of Bioinformatics, Fribourg, Switzerland; University of California San Francisco, UNITED STATES

## Abstract

Understanding how the nervous system bridges sensation and behavior requires the elucidation of complex neural and molecular networks. Forward genetic approaches, such as screens conducted in *C*. *elegans*, have successfully identified genes required to process natural sensory stimuli. However, functional redundancy within the underlying neural circuits, which are often organized with multiple parallel neural pathways, limits our ability to identify ‘neural pathway-specific genes’, i.e. genes that are essential for the function of some, but not all of these redundant neural pathways. To overcome this limitation, we developed a ‘forward optogenetics’ screening strategy in which natural stimuli are initially replaced by the selective optogenetic activation of a specific neural pathway. We used this strategy to address the function of the polymodal FLP nociceptors mediating avoidance of noxious thermal and mechanical stimuli. According to our expectations, we identified both mutations in ‘general’ avoidance genes that broadly impact avoidance responses to a variety of natural noxious stimuli (*unc-4*, *unc-83*, and *eat-4*) and mutations that produce a narrower impact, more restricted to the FLP pathway (*syd-2*, *unc-14* and *unc-68*). Through a detailed follow-up analysis, we further showed that the Ryanodine receptor UNC-68 acts cell-autonomously in FLP to adjust heat-evoked calcium signals and aversive behaviors. As a whole, our work (i) reveals the importance of properly regulated ER calcium release for FLP function, (ii) provides new entry points for new nociception research and (iii) demonstrates the utility of our forward optogenetic strategy, which can easily be transposed to analyze other neural pathways.

## Author summary

One of the main functions of the nervous system is to process sensory information about the environment and produce useful behaviors. For example, potentially damaging stimuli are detected by specialized neurons called nociceptors and sensory information processed to trigger avoidance behaviors. The nociceptive functions are largely conserved from animals to human, but the underpinning molecular mechanisms are far from being completely understood. Mutating genes in model organisms and screening for impaired avoidance is a powerful way to identify new molecular factors in nociceptive pathways. However, the approach is limited by the robust nervous system architecture often presenting parallel pathways with backup functions. Here, we introduce an experimental approach in the *Caenorhabditis elegans* model that overcomes this intrinsic robustness and enables the recovery of mutations with a non-pervasive impact, but nevertheless important for the function of specific neural pathways. After mutation mapping and through a series of additional experiments, we could identify novel avoidance genes and highlight intracellular signaling mechanisms working in specific nociceptor neurons. Our approach can be transposed to analyze other neural pathways and bring a more comprehensive view of the molecular network that is used by the nervous system to bridge sensation and behavior.

## Introduction

**Fig 1:**
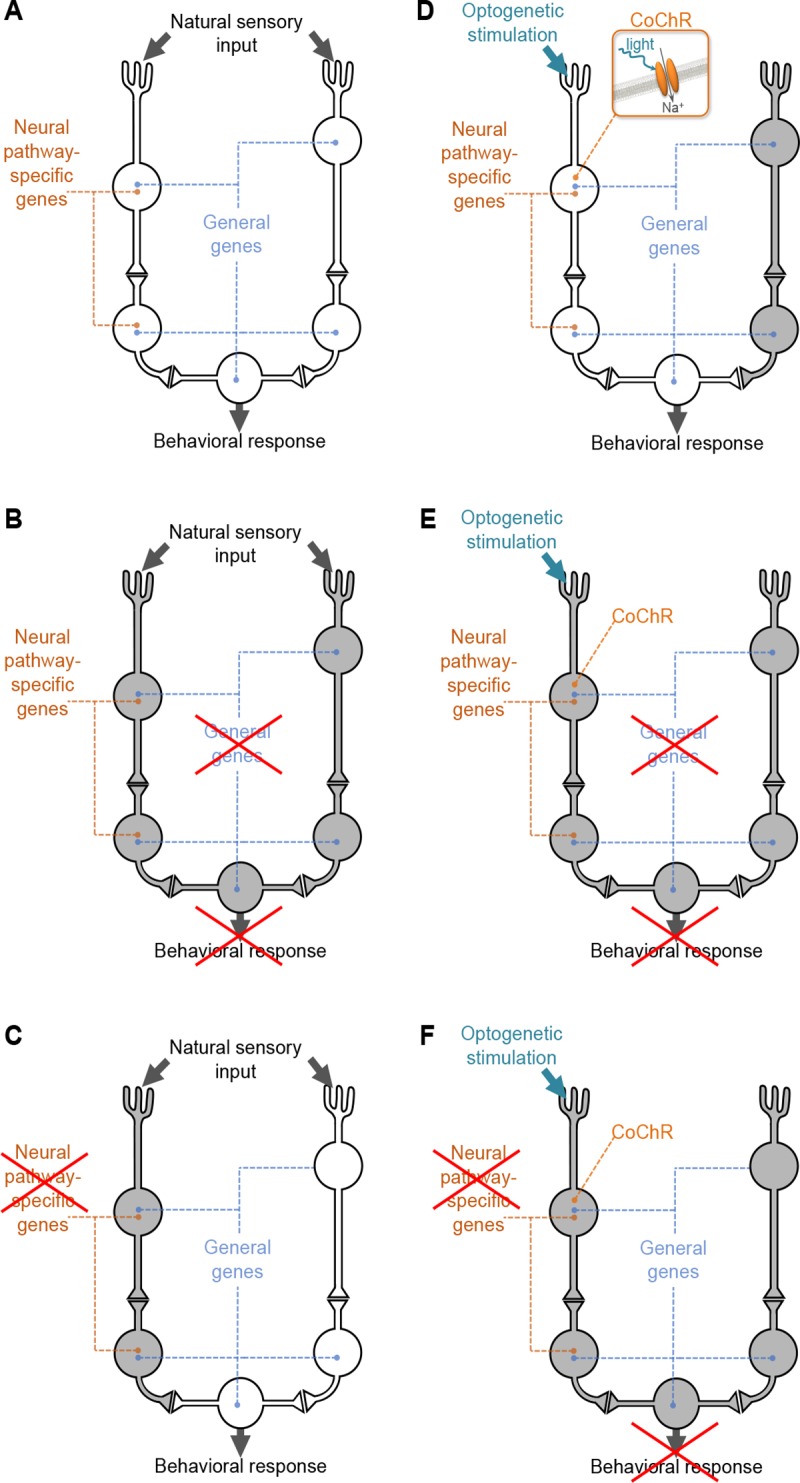
Principle of a forward optogenetics to identify pathway-specific and general genes controlling behavior. Scheme presenting a simple circuit showing degeneracy, with two parallel pathways conveying the sensory information, which later converge to produce a behavioral response. In each situation, active neural pathways are in white and inactive neural pathways are in grey. *Neural pathway-specific genes* are those genes required to support the function of only one pathway. *General genes* are those genes required to support the function of the two pathways. **A**: In wild type animals, a natural stimulus triggers a parallel activation of both pathways, producing a normal behavioral response. **B**: In mutant animals with disrupted *general genes*, both neural pathways are impaired. Thus, a natural stimulus cannot trigger a normal behavioral response. *General genes* are easily identified with classical screens using natural stimuli. **C**: In mutant animals with disrupted *neural pathway-specific genes*, one neural pathway is affected but the other one is intact. A natural stimulus can still trigger a normal behavioral response. *Neural pathway-specific genes* are difficult to identify with classical screens using natural stimuli. **D**: In transgenic animals expressing Channelrhodopsin in a specific neural pathway, light stimulation can be used to artificially activate this pathway and trigger a behavioral response that mimics the response to natural stimuli. The inset presents a schematic view of *Chloromonas oogama* Channelrhodopsin (CoChR), used in the present study. **E**: In the optogenetic transgenic background, mutations in *general genes* impair light-evoked behaviors, because all the pathways are disrupted, including the one activated by CoChR. **F**: In the optogenetic transgenic background, mutations in *neural pathway-specific genes* also impair light-evoked behaviors, because the CoChR-activated pathway is disrupted. This will cause a detectable phenotype and enable the identification of *neural pathway-specific genes*.

One of the main functions of the nervous system is to perceive environmental stimuli, process them, and produce appropriate behavioral responses to limit threat and damage to the organism and maximize survival and reproductive prospects. Efficient sensory behavior often entails complex neural circuits, connecting various types of neurons, such as sensory neurons required for stimuli sensation, interneurons implicated in signal processing and propagation and motor neurons driving muscular activity and behavioral outcome. Each type of neuron has specific functional properties conferred by specific sets of molecular components. Our understanding of the molecular framework involved in sensory behaviors (like chemo-, mechano-, osmo-, or thermo-sensation) has strongly benefited from classical genome-wide mutagenesis genetic screens such as those conducted in invertebrates [1–5]. With its well-defined behavioral repertoire, its small and fully mapped nervous system and the abundance of genetic tools, *C*. *elegans* is an ideal model for addressing the molecular, cellular and circuit basis of behavior. The analysis of *C*. *elegans* sensory behavior neural circuits revealed in many instances a high level of degeneracy[6–9], i.e. that they contain “structurally different components that can perform a similar function with respect to context”[10, page 14]. This functional redundancy between parallel neural pathways, which may exploit different sets of regulatory molecules, improves the robustness of behavioral responses, but at the same time limits the outcomes of forward genetic screens (Fig 1A, 1B and 1C). Indeed, conventional screens dealing with the response to natural stimuli tend to identify only ‘general’ genes that function in several neural pathways. In this case, a single mutation may disrupt more than one branch in the neural network and produce a phenotype that is sufficiently salient to be detected (Fig 1B). As a corollary, genes whose function is essential only for a subset of these pathways, which we will refer to as ‘neural pathway-specific’ genes, are more difficult to identify (Fig 1C). Indeed, when these ‘neural pathway-specific’ genes are mutated some alternative parallel pathways remain functional, resulting in little or no phenotypic impact. Identifying neural pathway-specific genes is nevertheless essential to obtain a comprehensive understanding of the molecular mechanisms underpinning sensory-behaviors.

We present here a forward optogenetics approach, which allows the identification of both ‘general’ and ‘neural pathway-specific’ genes. The principle is to selectively activate a target neural pathway using optogenetics rather than using natural stimuli, creating a simplified behavioral paradigm (Fig 1D). In this context, a mutagenesis screen can identify both ‘general’ (Fig 1E) and ‘pathway-specific’ genes (Fig 1F). As a proof of feasibility, we applied forward optogenetics to investigate the FLP neural pathways. The two FLP head neurons of *C*. *elegans* are polymodal nociceptors implicated in the sensation of harsh mechanical stimuli and noxious heat [11–13]. Selective optogenetic activation of FLP is sufficient to trigger a reversal, an avoidance response where animals moving forward switch to backward locomotion. Ablation of FLP neurons yields only a moderate reduction in the response to harsh touch [12] and noxious heat [11, 13], highlighting a partial redundancy with other neural pathways. Other sensory neurons known to detect anterior mechanical stimuli include ALM, AVM, ADE, CEP, OLQ and ASH, while those known to respond to thermal stimuli are AFD, AWC, and ASI [14, 15]. S1 Fig summarizes the known synaptic network that connects thermo- and mechano-sensory neurons to downstream interneurons and motor neurons controlling backward locomotion. Previous studies have identified genes expressed in FLP as candidates for mechanosensory and thermosensory transduction [12, 13, 16, 17], as well as for the modulation of noxious heat avoidance behavior according to past thermal experience [18]. However, despite this body of work, our knowledge on the molecular players controlling the function of FLP and downstream circuits is still very incomplete. Together with the high potential level of functional redundancy with parallel neural pathways, this has made FLP an interesting and challenging case to test the proposed forward optogenetic approach.

We present here the identification of genes necessary for the proper functioning of the FLP nociceptive pathway. Many of them had not previously been associated with nociception. According to our expectations, we highlight mutations that broadly impact avoidance responses to a variety of noxious stimuli, as well as mutations that produce a narrower impact, more restricted to the FLP pathway. Analyzing in details one mutant in the latter category, we uncover the cell-autonomous role of the Ryanodine Receptor UNC-68 in controlling the dynamic range of intracellular calcium signaling response to noxious heat in FLP, and resulting aversive behaviors. Overall, our work (i) demonstrates the feasibility and utility of a neuron-specific unbiased forward optogenetic approach and (ii) brings new insight on the molecular mechanism controlling aversive behaviors.

## Results

### Optogenetic screen design

**Fig 2:**
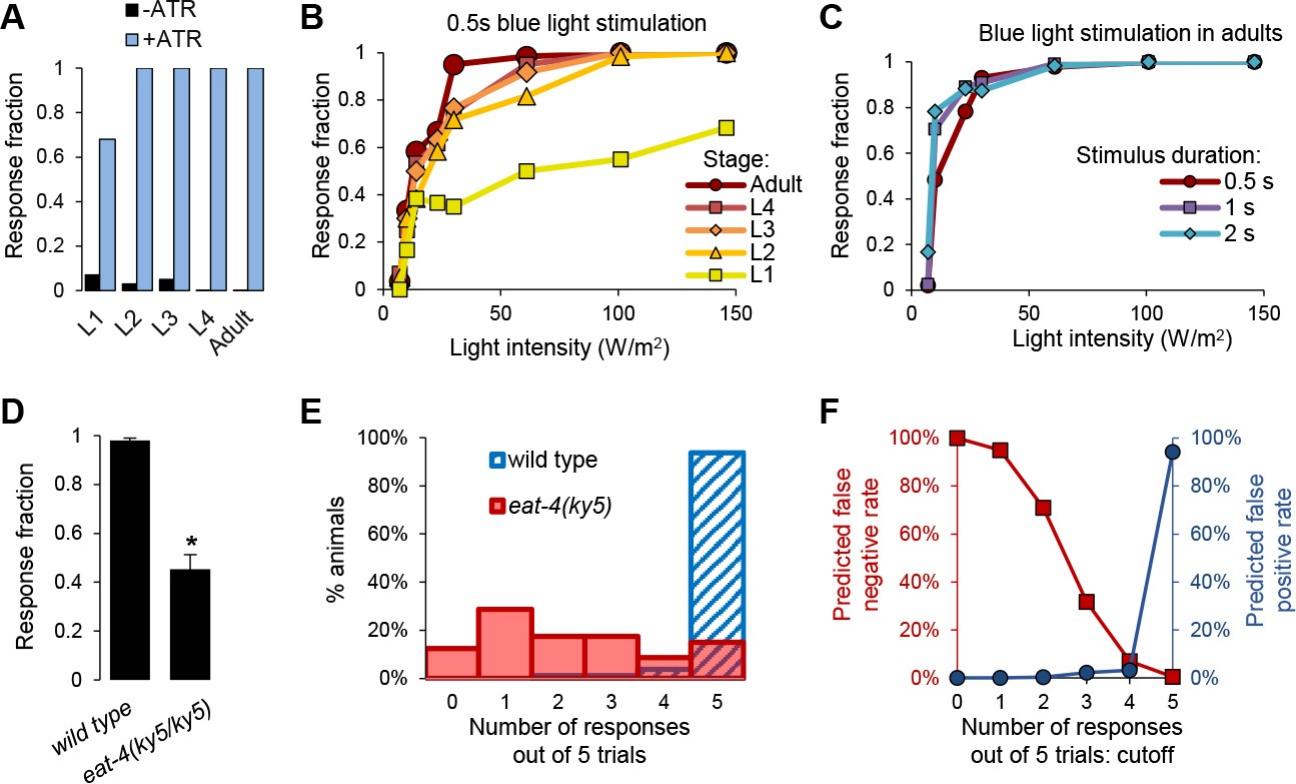
Determination of the screen conditions to identify genes required for FLP-evoked reversals. **A**: Comparison of reversal induction between *[FLP*::*CoChR]* animals grown in the absence (-ATR) or the presence (+ATR) of all-*trans-*retinal (ATR) and stimulated with 146 W/m^2^. (*n = 60* animals per condition). **B**: Light dose response curves in *[FLP*::*CoChR]* animals during their development from L1 larvae to adults. Each animal (*n = 60* per condition) was stimulated once for 0.5 s at the indicated light intensities. **C**: Light dose response curves in *[FLP*::*CoChR]* adult animals as in **B**, with varying light stimuli durations. **D**: Fraction of trials triggering a reversal response in wild type (*[FLP*::*CoChR]* background) and *eat-4(ky5)* mutants used as a positive control. Each animal was stimulated five times. *, *p* < .01 by Student’s *t-*test. **E**: Distribution of the number of responded trials from the experiment in **D**. Most wild type animals respond to 5 out of 5 trials, while the response rate is widespread in the *eat-4* mutant. **F**: Predicted false positive and false negative rates based on the comparison in **E**, according to different possible response cutoffs. A cutoff of “3 or less responded trials out of 5” was chosen to retain mutants during the initial screen.

In order to identify genes involved in FLP-mediated avoidance behavior, we developed a mutagenesis screen that looks for mutations impairing the behavioral response to FLP-specific optogenetic stimulations. Light stimuli are very convenient: individual animals can be stimulated rapidly and with extreme accuracy in terms of intensity and duration. Furthermore, unlike natural noxious stimuli, light stimuli are non-damaging for the animals. We used a previously established integrated transgenic line expressing the *Chloromonas oogama* channelrhodopsin (CoChR) in FLP and producing acute reversal behaviors in response to short light stimuli [19]. Blue light stimuli as high as 146 W/m^2^ triggered reversals when *[FLP*::*CoChR]* animals were grown in the presence of the all-*trans-*retinal (ATR) co-factor, but failed to do so in the absence of ATR (Fig 2A). Therefore, the exquisite sensitivity of CoChR allows us to use light intensities below the threshold for natural responses to short-wavelength (UV/blue) light [19–22].

When establishing the screen conditions, three main parameters were adjusted to minimize the expected number of false positives and false negatives. First, we examined the impact of the animal’s developmental stage on the robustness of FLP-evoked reversal responses. We found that both the sensitivity and the maximal response to light increased during development from L1 to adult stage, with a major step from L1 to L2 and a second minor step from L4 to adult (Fig 2B). We choose to carry out the screen with first day adults, as they displayed the most robust response. Second, we analyzed the impact of the intensity and the duration of light stimuli. In adults, we could reach 100% responsiveness with 61 W/m^2^ light stimuli lasting 0.5 s (Fig 2B). Lengthening the stimuli to 1 or 2 s slightly modified the shape of the light-dose response curves (Fig 2C), but the same intensity was still required to saturate the response. In order to minimize false positive rate while being able to recover mutants producing a mild sensitivity reduction, we decided to use the shortest stimulus, with the lowest intensity yielding a saturated response (0.5 s at 61 W/m^2^). Third, we adjusted the number of stimuli delivered to each animal and the criterion for retaining a candidate mutant. Because FLPs are glutamatergic neurons, we choose an *eat-4* mutant, known to selectively affect the loading of vesicles with glutamate [23], as a potential positive control. When each animal was tested with 5 repeated stimuli, we could see a robust response in wild type and an impaired response in *eat-4(ky5)* (Fig 2D and 2E). In order to define an appropriate screening criterion, we varied the response cutoffs and examined the effect on the expected false positive rate, as well as on the expected false negative rate for candidate mutants behaving like *eat-4* (Fig 2F). We decided to retain mutants responding to less than four out of five repeated trials, which represented a tradeoff between the false positive rate (~2%) and the false negative rate (~32%). With this criterion, we also estimated that we should recover the majority of mutants with a response rate partially reduced to ~68% of that in wild type.

### Identification of new FLP-mediated avoidance mutants

**Fig 3:**
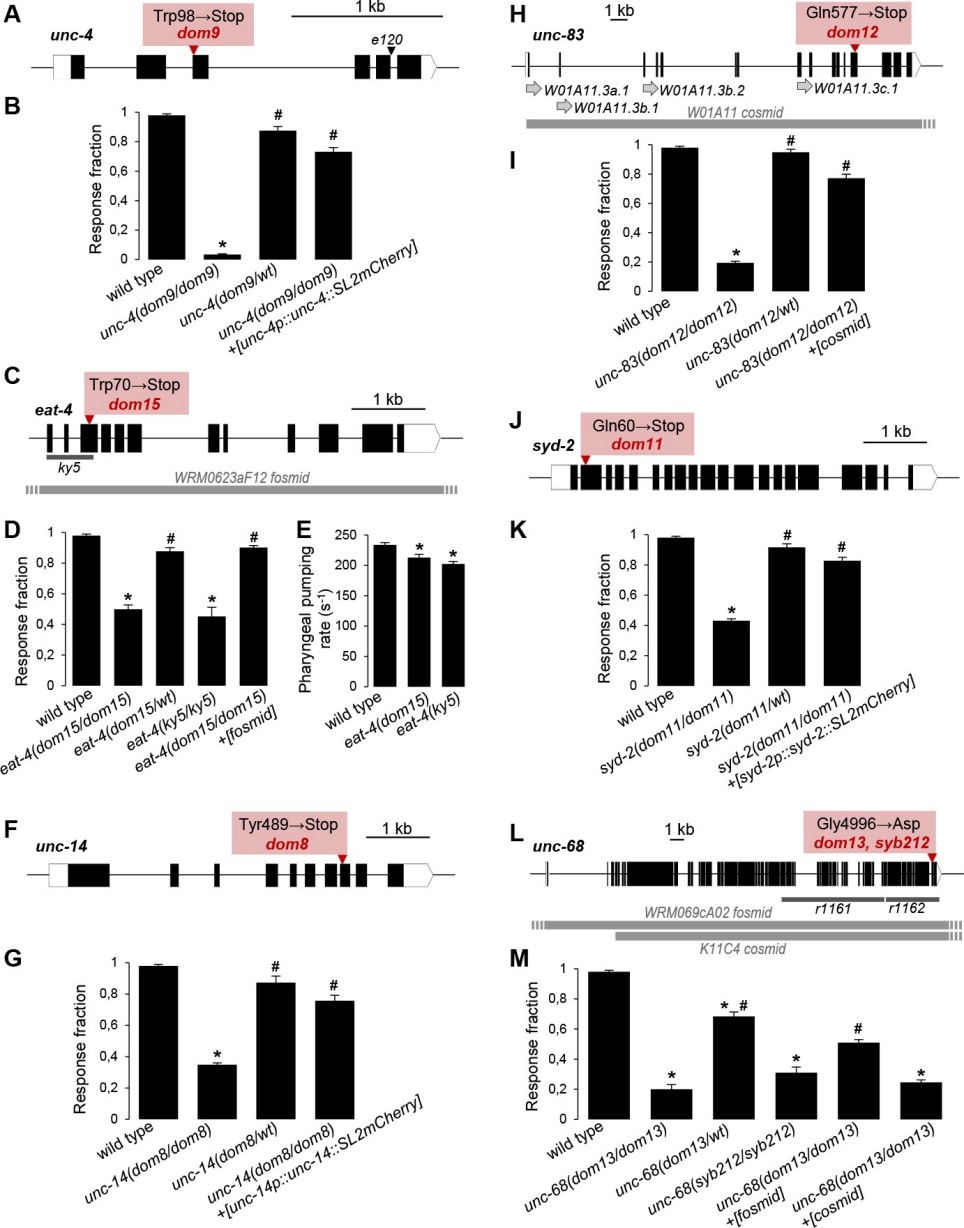
Mutations impairing FLP-evoked reversals. **A**, **C**, **F**, **H**, **J**, **L**: Scheme representing new mutations recovered from the screen for defects in reversals induced by optogenetic activation of FLP (red box). Genes are schematized with exons (black boxes) and untranslated regions (white boxes). Additional alleles used in the present study are also depicted, as well as genomic regions contained in cosmids and fosmids (horizontal grey bars). Grey arrows indicate alternative transcriptional starts in the *unc-83* gene (**H**). *syb212* (generated by genome editing) encodes the same UNC-68(G4996D) mutation as *dom13* (**M**). **B**, **D**, **G**, **I**, **K**, **M**: Fraction of blue light stimulation trials (61 W/m^2^) triggering a reversal response in adult *[FLP*::CoChR*]* animals. Results are presented as averages (bars) and SEM (error bars); *n*≥40 animals, each tested five times. **E**: Pharyngeal pumping rate assessed in adult animals of the indicated genotypes. Results are presented as averages (bars) and SEM (error bars). *n*≥40 animals, each scored over 20 s. One way ANOVAs indicated significant genotype effects in every situation. *, *p <* .01 versus wild type; #, *p* < .01 versus homozygote mutant, by Bonferroni post-hoc tests.

In total, the progeny of 2460 mutagenized F1 genotypes was screened for defective FLP-evoked avoidance behavior with the criteria described above. We recovered and mapped six mutants with a stable inherited phenotype: *dom8*, *dom9*, *dom11*, *dom12*, *dom13* and *dom15*. Five mutations (*dom8*, *9*, *11*, *12* and *15*) were recessive and one (*dom13*) was semi-dominant, with heterozygote animals presenting a mild defect in FLP-evoked reversals. Given the behavioral nature of the screen and the necessity to keep the optogenetic transgene, we decided to systematically employ Variant Discovery Mapping (VDM), the advantages of which are discussed in [24]. As expected, this method allowed to us to accurately pinpoint the location of each causative mutation. For each allele, we identified and validated candidate genes centered in the mapping interval that carried EMS-compatible mutations (S1 Table, Fig 3). The five recessive mutations were nonsense mutations and the semi-dominant mutation was a missense mutation, as described below.

#### unc-4(dom9)

VDM placed the *dom9* mutation on Chr II, likely between 5–11 Mb, with the highest mapping peak between 9–11 Mb (S2A Fig). Within the larger region, whole genome sequencing combined with subtraction of parental strain (DAG356) variants identified protein coding variants in four genes. One of them is a G to A transition in the *uncoordinated-4* (*unc-4*) Homeobox protein gene, introducing a premature Stop codon (W98Stop, Fig 3A) and is predicted to remove most of the Homeobox domain. Loss of *unc-4* function leads to miss-wired locomotion circuit and prevent the execution of backward movements mediated by AVA command neurons [25]. The FLP-evoked avoidance deficit in *unc-4(dom9)* was rescued in *[unc-4p*::*unc-4*::*SL2mCherry]* transgenic animals expressing *unc-4* cDNA under the control of a 2 kb *unc-4* promoter region (Fig 3B). These observations indicate that *unc-4(dom9)* is a loss-of-function allele and that UNC-4 is required for the FLP-evoked reversal response, which is consistent with a general impairment in backward locomotion[25].

#### eat-4(dom15)

VDM placed the *dom15* mutation on Chr III, between 8–10 Mb. This region contains three protein coding variants, one of which is a G to A transition allele in the EAT-4/VGLUT transporter gene *eat-4*, introducing a Stop codon at position 70 and encoding a truncated protein lacking all twelve predicted transmembrane domains (Fig 3C). The FLP-evoked avoidance defect in *eat-4(dom15)* was rescued with a WRM0623aF12 fosmid containing the *eat-4* genomic region (Fig 3D). Furthermore, this mutant had phenotypes similar to those of the reference loss-of-function mutant *eat-4(ky5)*, showing reduced FLP-evoked reversal response (Fig 3D) and pharyngeal pumping rate (Fig 3E). These data indicate that *eat-4(dom15)* most likely represents an *eat-4* loss-of-function, and confirm that the EAT-4/VGLUT transporter is required for FLP-evoked avoidance behavior.

#### unc-14(dom8)

VDM placed the *dom8* mutation on Chr I, between 6–8 Mb (S2B Fig). This region contains protein coding variants in seven genes, one of which is a T to A transversion introducing a premature stop (Y489Stop, Fig 3F) in the *uncoordinated-14* (*unc-14*) gene. *unc-14* encodes a RUN domain (for RPIP8, UNC-14, and NESCA) protein expressed in both neuronal and non-neuronal tissues, which plays a role in axonogenesis and axon guidance [26–28]. The FLP-evoked avoidance deficit in *unc-14(dom8)* was rescued in *[unc-14p*::*unc-14*::*SL2mCherry]* transgenic animals expressing *unc-14* cDNA under the control of a 2 kb *unc-14* promoter region, confirming that intact UNC-14 is necessary for this behavior (Fig 3G). Also, we noticed that *unc-14(dom8)* animals tended to coil, as described for other *unc-14* loss-of-function mutants [29]. These data suggest that *unc-14(dom8)* is a loss-of-function allele and that intact *unc-14* function is required for FLP-evoked reversal responses.

#### unc-83(dom12)

VDM placed the *dom12* mutation on Chr V, between 6–8 Mb (S2C Fig). This region contains protein coding variants in four different genes. One mutation is a C to T transition introducing a premature Stop codon (Q577Stop, Fig 3H) in the *uncoordinated-83* (*unc-83*) gene. This mutation is predicted to truncate the four *unc-83* isoforms made through alternative transcription start (W01A11.3a, W01A11.3b.1, W01A11.3b.2 and W01A11.3c; as defined in wormbase version WS264, Fig 3H). UNC-83 is a KASH domain (for Klarsicht, ANC-1, Syne Homology) protein playing a role in nuclear migration and development [30–33]. The FLP-evoked avoidance was rescued in *unc-83(dom12)* mutants with a W01A11 cosmid covering the exonic region for the four *unc-83* isoforms, but lacking the promoter region for W01A11.3a.1 (Fig 3I). These data indicate that at least some *unc-83* isoforms are required to support a normal FLP-evoked reversal response.

#### syd-2(dom11)

VDM placed the *dom11* mutation on Chr X, between 9–11 Mb (S2D Fig). This region contains protein coding variants in three different genes. One mutation is a C to T transition introducing a premature STOP (Q60Stop, Fig 3J) in the *synapse defective-2* (*syd-2*) gene encoding the SYD-2/Liprin-α presynaptic scaffold protein that is widely expressed in the nervous system and involved in dense core vesicle trafficking and in the formation and stability of pre-synaptic structures [34–36]. This mutation is supposed to remove all the functionally-relevant coiled-coil regions [37]. The FLP-evoked avoidance defect in *syd-2(dom11)* mutants was rescued in *[syd-2p*::*syd-2*::*SL2mCherry]* transgenic animals expressing *syd-2* cDNA under the control of a 2 kb *syd-2* promoter region (Fig 3K). These data indicate that intact SYD-2 is required for a normal FLP-evoked reversal response.

#### unc-68(dom13)

VDM placed the *dom13* mutation on Chr V, between 6–9 Mb (S2E Fig). This region contains three protein coding variants in two different genes. One of these is a G to A mutation in the Ryanodine Receptor (RyR) gene *unc-68* that causes a G4996D mutation in the protein (Fig 3L). RyRs are calcium-activated calcium channels at the membrane of the endoplasmic/sarcoplasmic reticulum (ER/SR)[38] and expressed in neurons and muscle in *C*. *elegans* [39]. To verify that this *unc-68(dom13)* mutation was the cause of the FLP-mediated avoidance defect, we engineered the same mutation with CRISPR/Cas9 genome editing. We observed a similar phenotype in the resulting *unc-68(syb212)* mutant (Fig 3M). Furthermore, the avoidance phenotype in *unc-68(dom13)* was partially rescued in transgenic animal lines made with a fosmid covering the whole *unc-68* gene region. Control transgenic animals made with a cosmid that did not cover the full *unc-68* coding sequence failed to rescue *unc-68(dom13)*. These results establish that the *unc-68(dom13)* mutation is indeed responsible for the FLP-evoked reversal response defect.

**Fig 4:**
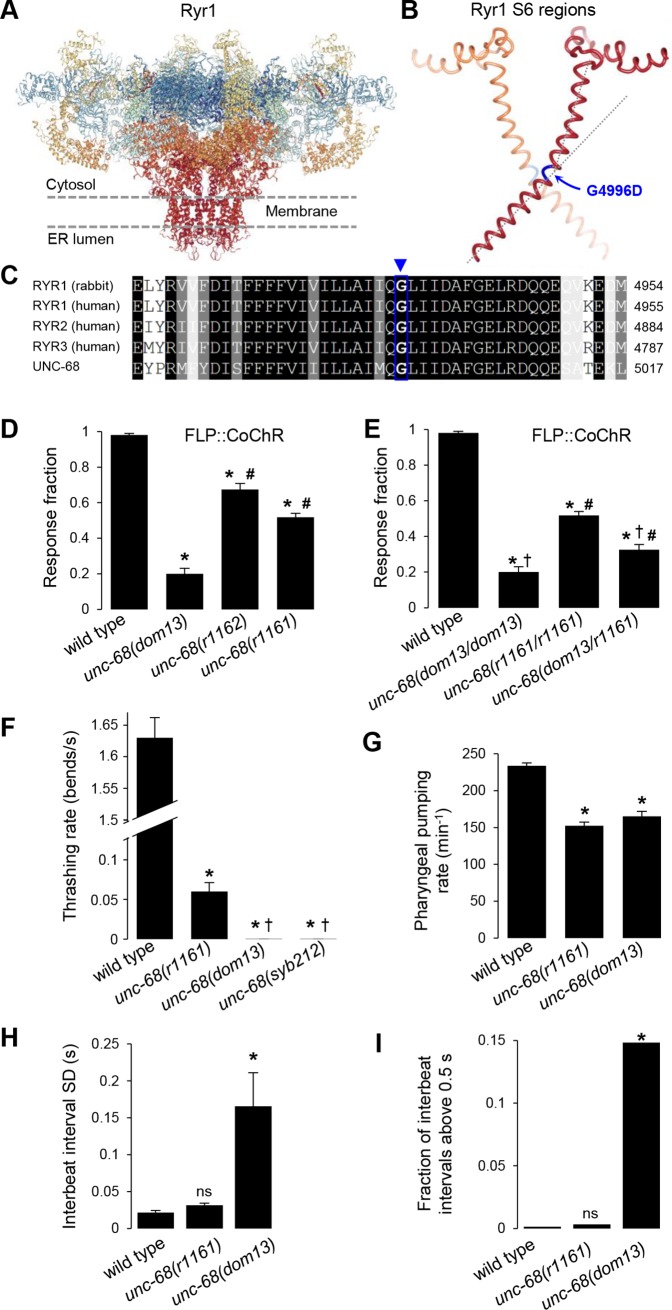
The Ryanodine receptor G4996D mutation in the conserved S6 region is a gain-of-function. **A**: Overview of the Ryanodine receptor Ryr1 structure (rabbit, pdb: 5TB0), composed of four protomers. **B**: Close-up view of the Ryr1 S6 region and localization of the glycine hinge G4996D mutation in *unc-68(dom13)* allele. Only two facing protomers are depicted. **C**: Primary structure alignment across RyRs showing high conservation, including the hinge glycine in the S6 region (blue arrow). **D**, **E**: FLP-evoked reversal analysis in optogenetically stimulated adult *[FLP*::CoChR*]* animals carrying wild type, gain-of-function *(dom13)*, and loss-of-function *(r1161)* alleles of *unc-68*, showing a stronger impact for the gain-of-function than for the loss-of-function allele. Bars indicate the average fraction of trials causing a reversal response, with SEM (error bars); *n*≥40 animals, each tested five times. *, †, #, *p* < .01 versus wild type, *r1161* and *dom13* homozygotes, respectively, by Bonferroni post-hoc tests. **F**: Swimming behavior analysis in wild adult animals and different *unc-68* homozygote mutants, showing a more dramatic impact of the gain-of-function alleles *dom13* and *syb212*. *, †, *p* < .01 versus wild type and *unc-68(r1161)*, respectively, by Bonferroni post-hoc tests. **G**, **H**, **I**: Pharyngeal pumping analyses in adult wild type animals and different *unc-68* mutants. *n*≥30 animals. **G:** Average pumping rate comparison (bars, +/- SEM) showing defects in both loss- and gain-of-function mutants. *, *p* < .01 versus wild type by Bonferroni post-hoc tests. **H**: Interbeat interval comparison showing stronger variability in the *unc-68(dom13)* gain-of-function mutant. Bars indicate the average standard deviations (SD) of the interbeat interval distribution for each animal, with SEM as error bars. *, *p* < .01 versus wild type by Dunnett’s tests. ns, not significant. **I**: Interbeat interval comparison showing frequent pumping pauses in the *unc-68(dom13)* gain-of-function mutant. Bars indicate the fractions of abnormally long interbeat intervals (longer than 0.5 s).*, *p* < .001 by Fisher’s exact tests.

Next, we wanted to determine if *unc-68(dom13)* is a loss- or a gain-of-function mutation. The semi-dominant behavior of this allele (Fig 3M), as well as the change in amino acid it produces, led us to favor the gain-of-function hypothesis. Indeed, the G4996D mutation targets an essential and conserved residue (G4934 in rabbit), which, in the mammalian RYR1, enables the formation of a hinge into the S6 helix of the pore-forming region (Fig 4A, 4B and 4C). Modifying this helix can change the opening or closing properties of the channel. We provide four experimental evidences suggesting that *unc-68(dom13)* is indeed a gain-of-function mutation. First, we compared the impact of *unc-68(dom13)* with that of recessive null mutations previously identified: *unc-68(r1161)* and *unc-68(r1162)*. Whereas homozygote mutants for either null allele displayed a significant reduction in the FLP-evoked avoidance behavior, this effect was significantly less pronounced than that in *unc-68(dom13)* mutants (Fig 4D). Second, we examined FLP-evoked reversals in hemi-zygote animals carrying one copy of the null *unc-68(r1161)* allele and one copy of the *unc-68(dom13)* allele. If the *dom13* allele was a loss-of-function, one would expect to see a similar or less dramatic phenotype than that in null homozygotes. Conversely, if it was a gain-of function allele, the phenotype should be more severe than in the null homozygotes. Our results are in agreement with the latter model, with *unc-68(r1161/dom13)* having a stronger reduction in FLP-evoked avoidance than the null (Fig 4E). Third, we compared the impact of different alleles of *unc-68* on worm swimming. Maryon and collaborators [40] previously showed that *unc-68* null mutants swim with a significantly reduced thrashing rate, and that an even stronger reduction is produced in wild type animals treated with ryanodine at a concentration blocking the channel in an open state [41] and causing muscular hypercontraction and paralysis. As if *unc-68(dom13)* was encoding an over-activated channel, we found that this mutant had a dramatic swimming deficit, more pronounced than that in the *unc-68(r1161)* null (Fig 4F). Fourth, we compared the impact of *unc-68* mutations on pharyngeal pumping. As previously shown [42], *unc-68(r1161)* animals had a reduced pharyngeal pumping rate (Fig 4G). The average pumping rate was similarly reduced in *unc-68(dom13)* (Fig 4G), but for a different reason than in the null mutants. The *r1161* mutation reduced the average pumping rate by homogeneously extending the inter-beat intervals. In contrast, the *dom13* mutation caused arrhythmia with a significantly higher standard deviation of the inter-beat interval distribution (Fig 4H) and an increased fraction of inter-beat intervals above 0.5 s (Fig 4I).

Collectively, these results suggest that *unc-68(dom13)* is a gain-of-function mutation, most likely encoding a deregulated channel. Furthermore, since both gain- and loss-of-function mutations in *unc-68* impair nociceptive behavioral responses, these results also suggest that an appropriate level of UNC-68 activity is essential for FLP-evoked avoidance.

### New mutations affect the responses to natural stimuli detected by FLP

The mutations identified in our screen impair avoidance responses triggered by an optogenetic activation of FLP, which is an artificial situation. We next addressed whether these mutations could also impact the animal response to natural stimuli, starting with stimuli known to be detected by FLP.

#### Head harsh touch

**Fig 5:**
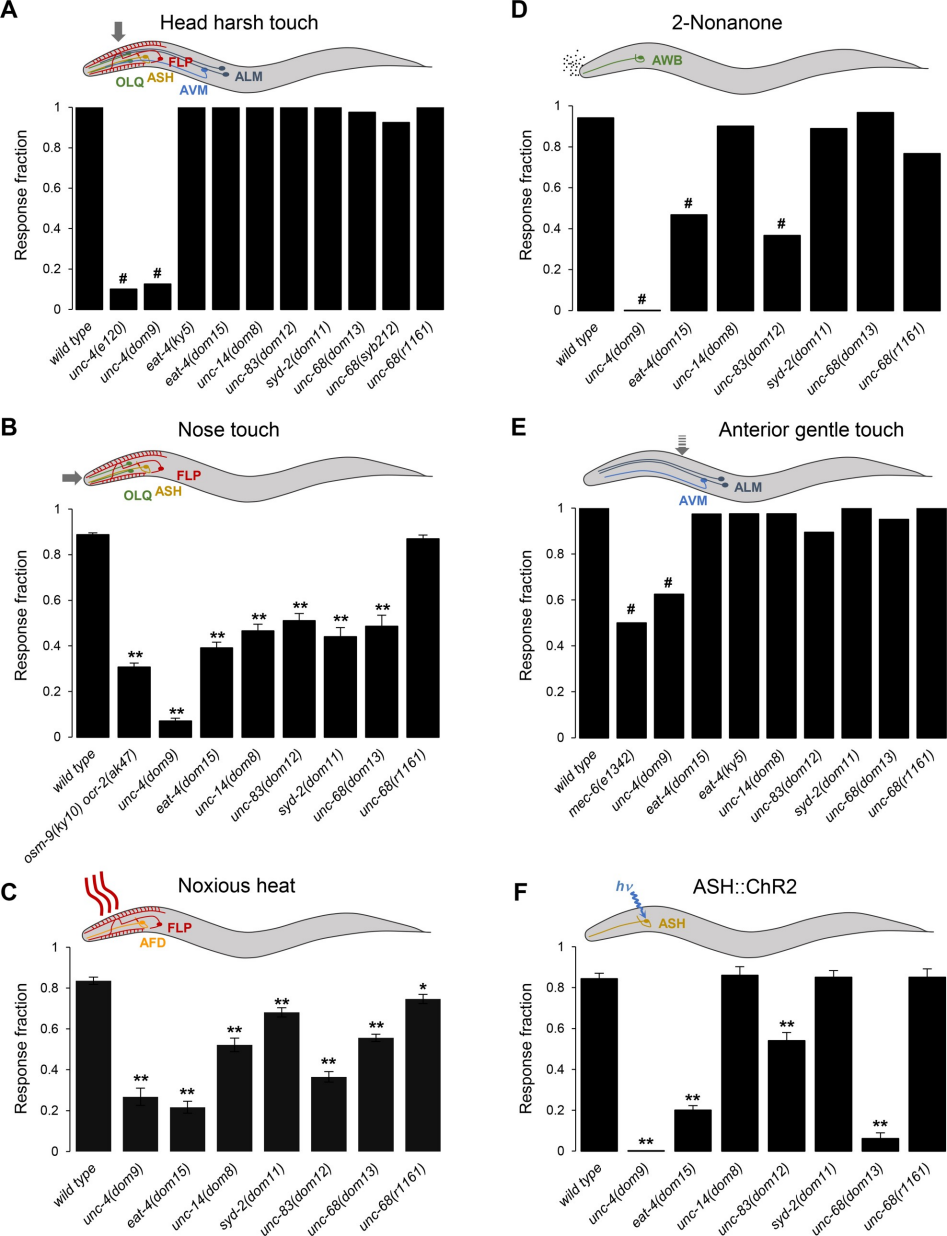
Gene-specific patterns of aversive behavior impairments in mutants. Quantification of reversal behavior induced by stimuli detected dependently (**A**, **B**, **C**) and independently (**D**, **E**, **F**) of FLP. Stimuli were head harsh touch (**A**), nose touch (**B**), noxious heat (**C**), the chemical repellent 2-nonanone (**D**), gentle anterior touch (**E**), and optogenetic activation of the ASH nociceptor neuron in an *[ASH*::*ChR2]* transgenic background (**F**). Schemes at the top of each panel illustrate the stimuli and the sensory neurons involved. (**A**, **D**, **E**) Bars indicate the fractions of trials triggering a response. *n≥* 40 animals, each tested with a single stimulus. #, *p <* .01 versus wild type by Bonferroni-corrected repeated Fisher’s exact tests. (**B**, **C**, **F**) Bars indicate the average fractions of trials triggering a response (with SEM as error bars). *n≥* 40 animals, each tested with five (C, F), or ten (B) stimuli. *, *p <* .05 and **, *p <* .01 versus wild type by Dunnett’s tests.

We quantified harsh touch-evoked avoidance behavior by vertically poking animals with a platinum wire on the head, a region innervated by FLP but also by several additional mechanosensory neurons (ASH, CEP, OLQ, ADE, ALM and AVM). In forward-moving wild type adults, head poking induced robust reversal responses (Fig 5A). Consistent with a general impairment of backward locomotion, the behavioral response to such stimuli was almost entirely abolished in *unc-4(dom9)*, as well as in *unc-4(e120)*, another loss-of-function allele. Conversely, the sensitivity to head poking in *eat-4*, *unc-14*, *unc-83*, *syd-2* and *unc-68* mutants was unaffected, which is in striking contrast with the strong impairment in optogenetically evoked FLP-specific responses (Fig 3). These results indicate that these five mutations (i) do not cause FLP-evoked avoidance defects though a general impairment of worm’s ability to produce reversal, (ii) leave intact the function of some mechano-nociceptive neural pathways, and (iii) would have been difficult to recover in a harsh touch-based screen.

#### Nose touch

We next assessed the mutant response in nose touch assays, in which crawling animals frontally hit an obstacle. This response is mediated by a narrower set of head mechanosensory neurons: ASH, FLP and OLQ. Mutants for all six genes displayed decreased nose touch responses (Fig 5B). Nose touched-induced reversals were almost abolished in *unc-4* mutants, an effect even stronger than that observed in a double *osm-9 ocr-2* mutant used as positive control in our assays [43, 44]. In contrast, the response decrease was only partial in *eat-4*, *unc-14*, *unc-83*, *syd-2* and *unc-68(dom13)* mutants and not significant in *unc-68(r1161)*. These results suggest that part of the nose touch response circuit remains functional in these mutants.

#### Noxious heat

We next examined noxious heat avoidance, known to be mainly mediated by AFD and FLP neurons. Animals were exposed to 4 s heat stimuli, which trigger robust reversal responses in wild type (Fig 5C). Noxious heat induced reversals were strongly impaired in *unc-4*, *eat-4* and *unc-83* mutants. A more partial heat avoidance defect was observed for *unc-14*, *syd-2* and *unc-68* mutants.

Collectively, results of these avoidance assays show that the mutations identified in our optogenetic screen also modify the responses to natural stimuli detected by FLP, and, this, to various degrees according to the mutation and the stimulus.

### Mutant response to FLP-independent sensory pathways

In order to determine whether the new mutations retrieved in our screen selectively affect the FLP pathway or the avoidance responses more generally, we analyzed mutant responses to additional stimuli triggering reversal responses, but that are detected independently of FLP.

#### 2-nonanone avoidance

First, we examined reversal responses induced by the chemical repellent 2-nonanone, which is detected by the chemoreceptor AWB [45]. Consistent with a general impairment in reversal, the response was abolished in the *unc-4(dom9)* mutant (Fig 5D). A partial reduction was observed for *eat-4* and *unc-83*, and no significant effects were observed for *unc-14*, *syd-2*, and either gain- or loss-of-function alleles of *unc-68*.

#### Anterior gentle touch

We next tested the response to anterior gentle touch stimuli, detected by ALM and AVM. To allow comparison with our harsh touch assay (Fig 5A), we scored each animal with a single gentle anterior stimulus. We observed a significant reduction in gentle touch-induced reversal responses in *unc-4* mutants, similar to that in *mec-6* mutants, used as a positive control (Fig 5E). In contrast, the gentle touch response rate was unaffected in *eat-4*, *unc-14*, *unc-83*, *syd-2*, and *unc-68* mutants, suggesting that these genes are dispensable for the ability of ALM and AVM to detect touch under our conditions.

#### ASH-mediated avoidance

The mutants have little or no impact on the nose touch-evoked response. The residual nose touch response in these mutants could be due to intact responsiveness of mechanosensory neurons functioning redundantly with FLP, such as ASH. To test whether the ASH pathway was affected in mutants, we assessed the impact of these mutations in transgenic animals in which ASH could be stimulated optogenetically (Fig 5F). The different mutations had various impacts on ASH-evoked reversals. Loss of *unc-4* or gain of *unc-68* function almost abolished ASH-mediated responses, loss of *eat-4* or *unc-83* function produced a partial reduction, whereas loss of *syd-2*, *unc-14* or *unc-68* function left intact the ASH-mediated response.

Taken together, and as visually summarized in S3 Fig, our results indicate varying degrees of stimulus-evoked reversal impairments across the new mutants recovered from the screen. On one extreme side, loss of *unc-4* has a very general and strong impact on reversal behaviors. On the other side, loss of *syd-2*, *unc-14* or *unc-68* produces a narrower impact, notably on FLP-dependent responses.

### New mutations do not affect FLP development and differentiation

**Fig 6:**
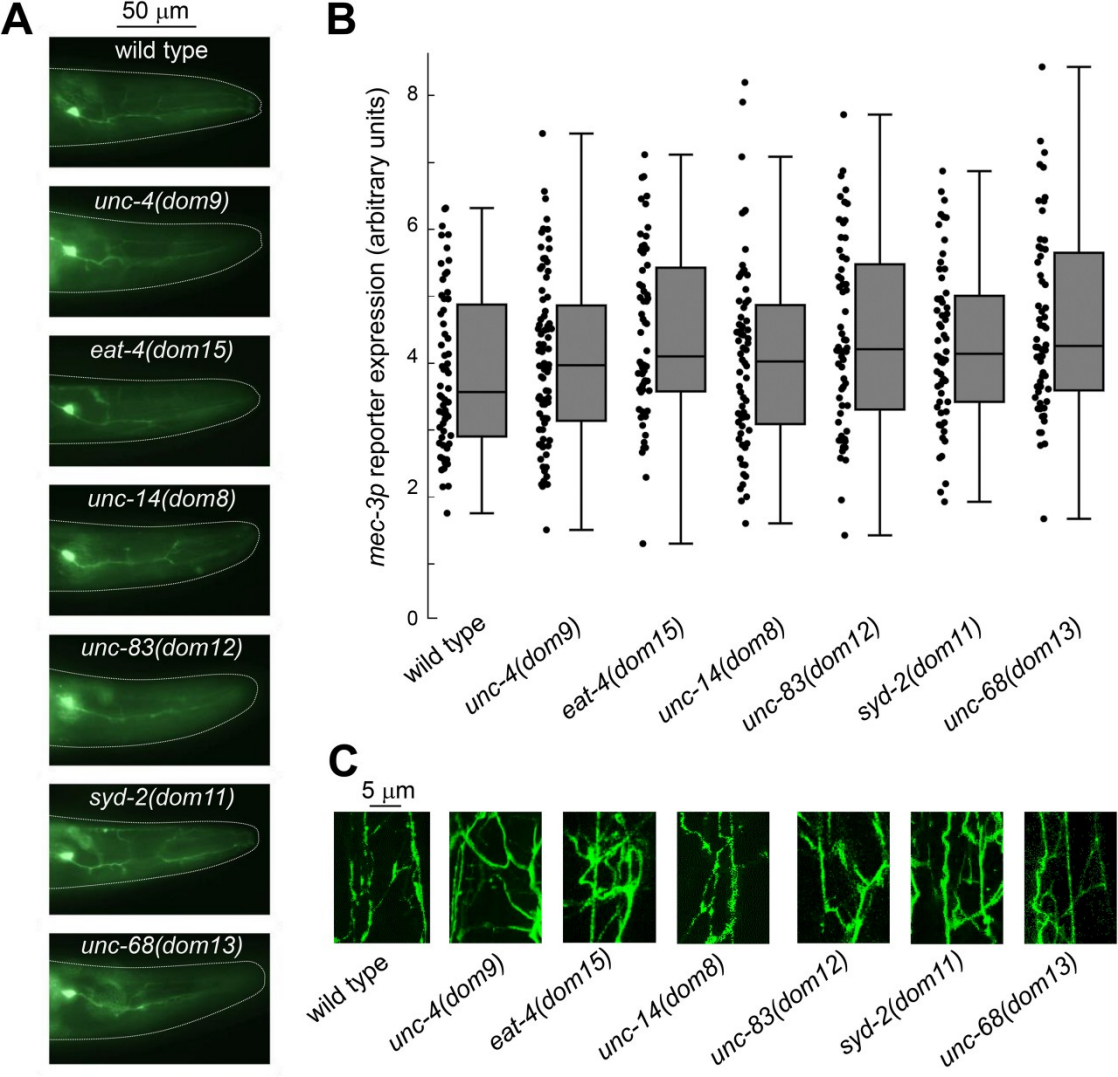
Intact FLP development in *unc-4*, *eat-4*, *unc-14*, *unc-83*, *syd-2*, *and unc-68* mutants. **A**: Epifluorescence micrographs of FLP neurons labelled with a *mec-3p*::*cmk-1*::*mNeonGreen* transgene, in wild type and indicated homozygote mutants. **B**: Quantification of the *mec-3p*::*cmk-1*::*mNeonGreen* reporter expression in FLP cell bodies, showing no difference in the transcription of this FLP differentiation marker between wild type and mutants (one way ANOVA, *p*>.01). Box plots and individual data points. *n≥*60 animals. **C**: Confocal image projections showing the complex FLP neurite arborization in wild type and mutant animals.

In order to better understand how our new mutations impact FLP-mediated behaviors, we next examined whether they affect FLP development or differentiation. Using a fluorescent reporter labeling FLP cytoplasm, we did not observe obvious anatomical defects in *unc-4(dom9)*, *eat-4(dom15)*, *unc-14(dom8)*, *unc-83(dom12)*, *syd-2(dom11)* and *unc-68(dom13)* mutants, all showing normal cell body positions and well-developed neurite arborizations that were similar to those in wild type animals (Fig 6A and 6C). We also quantified the expression of a transcriptional reporter for *mec-3*, which is a marker of FLP differentiation [46], and found no significant differences between the tested genotypes (Fig 6B). While we cannot exclude more subtle alterations, these observations suggest that proper *unc-4*, *eat-4*, *unc-14*, *unc-83*, *syd-2* and *unc-68* functions are dispensable for normal FLP development and differentiation.

### *eat-4*, *unc-14* and *syd-2*, but no *unc-4*, are required in FLP to control avoidance

In order to gain more insight on how the different genes identified in the screen affect the FLP pathway, we next conducted cell-specific rescue experiments in mutants. We restored the expression of *unc-4*, *eat-4*, *unc-14* and *syd-2* in FLP (and a few other neurons) by using transgenes fusing the *mec-3* promoter upstream of respective coding sequences (cds). The effects of these cell-specific rescue transgenes on FLP-evoked avoidance were compared to those of transgenes carrying the endogenous promoters for each gene.

**Fig 7:**
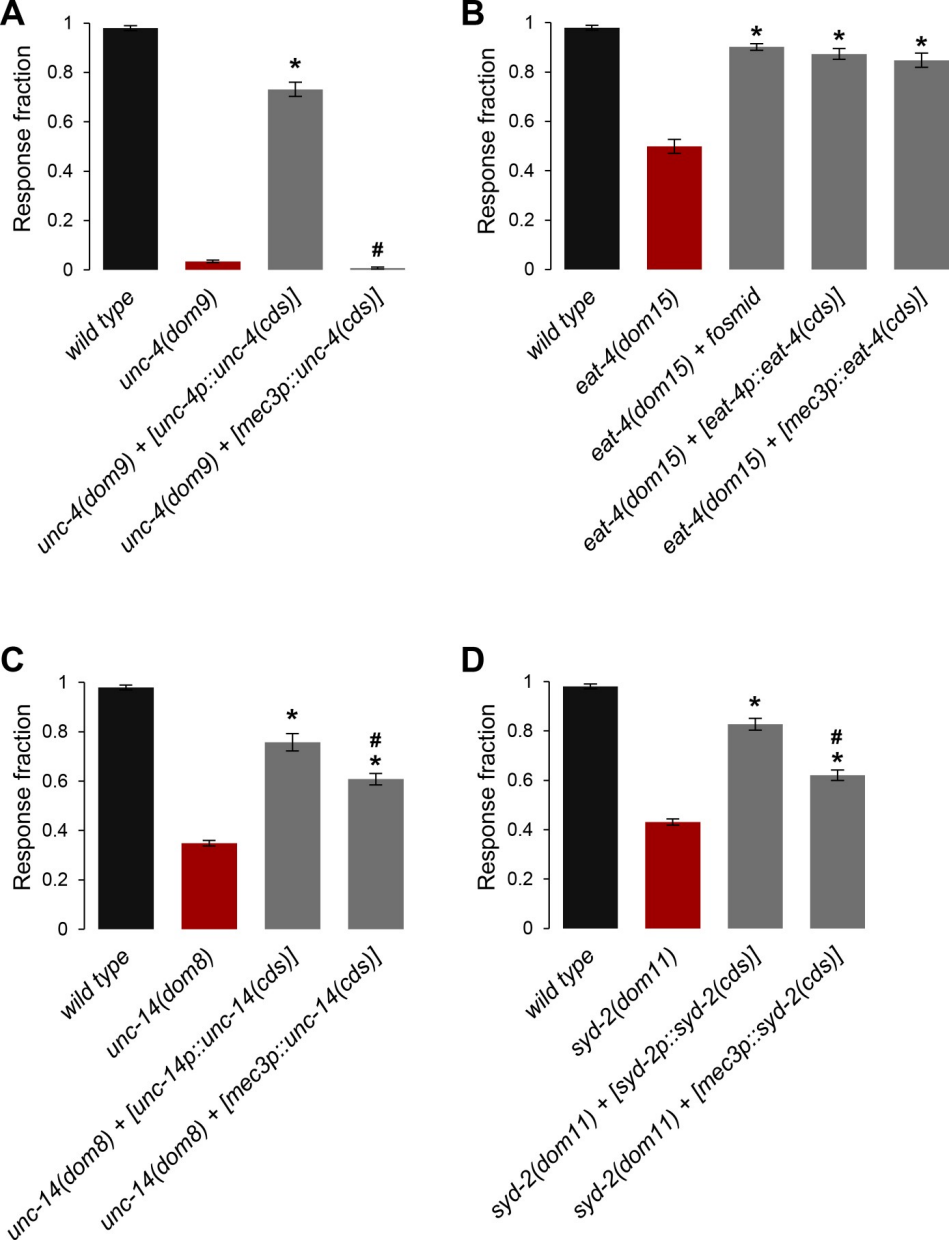
Tissue-specific rescue of *unc-4*, *eat-4*, *unc-14*, *and syd-2* mutations. **A**, **B**, **C**, **D**: Fraction of blue light stimulation trials (61 W/m^2^) triggering a reversal response in adult *[FLP*::CoChR*]* animals. Mutations and specific transgenes used for genetic rescue attempts are depicted below each plot. Results are presented as averages (bars) and SEM (error bars); *n*≥40 animals, each tested five times. One way ANOVAs indicated significant genotype effects in every situation. *, *p* < .01 versus mutant, #, *p <* .01 versus endogenous promoter rescue, by Bonferroni post-hoc tests.

The dramatic decrease in FLP-evoked avoidance in *unc-4(dom9)* mutation was almost fully rescued by the *[unc-4p*::*unc-4(cds)]* transgene, but left unaltered by the *[mec-3p*::*unc-4(cds)]*transgene (Fig 7A). *unc-4* expression in FLP is therefore not sufficient to rescue the mutant phenotype, suggesting that the expression of this gene is required in additional cells, most likely cells that are downstream of FLP.

In contrast, the decrease in FLP-mediated avoidance in *eat-4(dom15)* mutation was equally and markedly rescued by a *eat-4-*carrying fosmid, the *[eat-4p*::*eat-4(cds)]* transgene, and the *[mec-3p*::*eat-4(cds)]* transgene (Fig 7B). Thus, *eat-4* expression in FLP is sufficient to enable FLP-evoked avoidance and dispensable in other cells.

For *unc-14* and *syd-2*, FLP-specific rescue produced a partial rescue effect (Fig 7C and 7D). These results indicate that these genes at least partially act in FLP to control FLP-evoked avoidance. The fact that we obtained stronger rescue effects with respective endogenous promoters may reflect either the involvement of additional cells, or the fact that the *mec-3* promoter does not appropriately recapitulate the endogenous expression level or timing in FLP.

Collectively, these results suggest distributed places of action for the different genes recovered in our screen within the FLP neural pathway.

### UNC-68 functions cell-autonomously in FLP to control avoidance

In order to determine how UNC-68 could control FLP-evoked avoidance, we also wanted to determine its place of action. Because of the extreme size of this gene (>27 kb) and its coding sequence (>15 kb), we had to engage a different set of transgenic tools than the one used in the previous section for *unc-4*, *eat-4*, *unc-14*, and *syd-2*.

**Fig 8:**
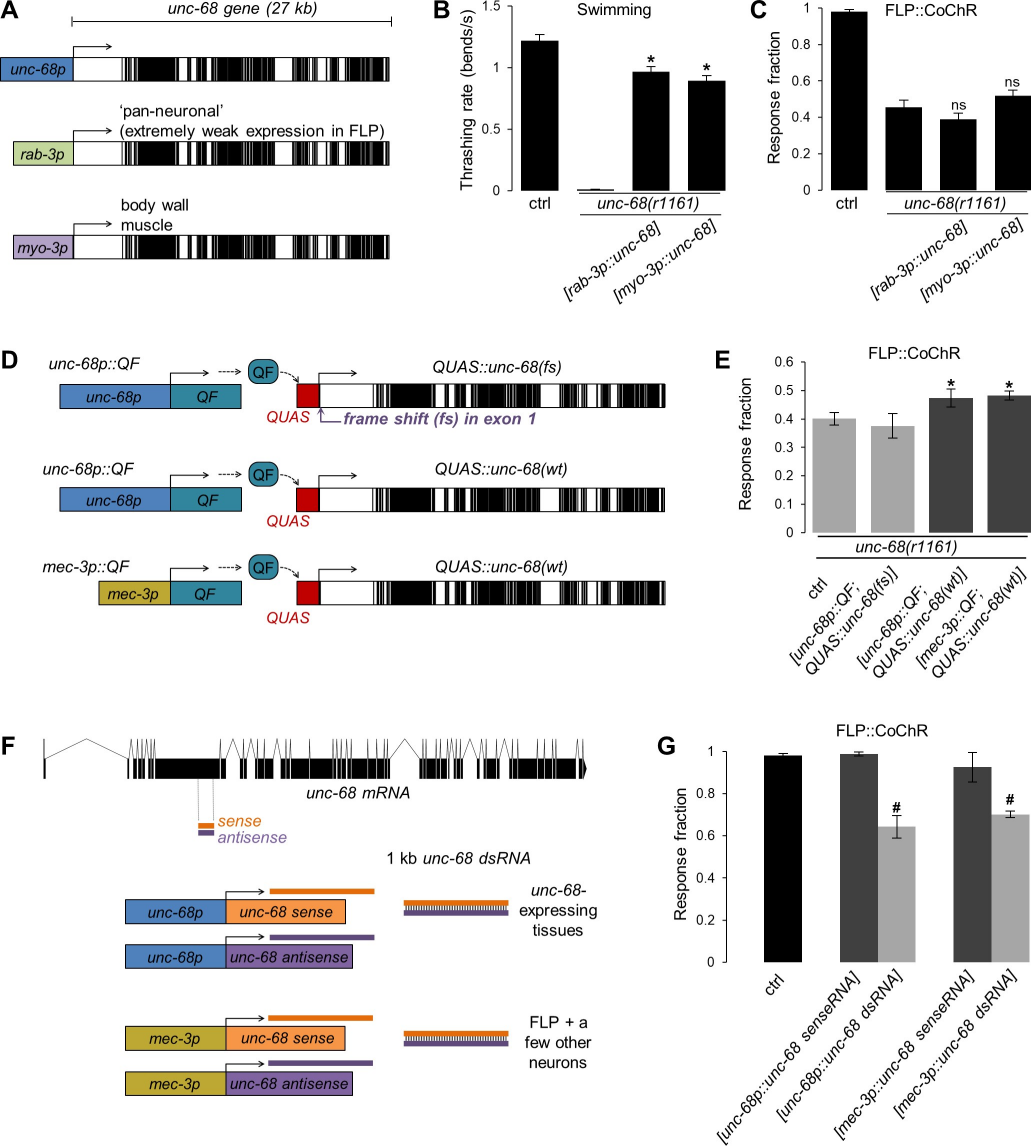
UNC-68 functions cell-autonomously in FLP to control reversal induction. **A**: Schematic of the transgenes used for muscle- and neuron-specific rescues of *unc-68*. Note that the *rab-3* promoter drives almost undetectable expression in FLP neurons (S4 Fig). **B, C**: Expression of *unc-68* with the *myo-3* or *rab-3* promoter is sufficient to rescue the swimming defect (**B**), but not the FLP-evoked reversal defect (**C**) in *unc-68(r1161)* mutants. *, *p* < .01; ns, not significant versus *unc-68(r1161)* control by Dunnett’s tests. *n*≥38 animals (**B**) and *n*≥80 animals (**C**). Bars indicate averages (+/- SEM). **D**: Schematic of the QF/QUAS two-component system used to drive *unc-68* expression in FLP with the *mec-3* promoter. Different plasmids (left) are used to drive the expression of the transcription factor QF in the desired tissue. QF can then bind to the QUAS promoter in a co-injected fosmid to drive the expression of a functional *unc-68(wild type)* transgene or a non-functional *unc-68(fs) (frame shift)* transgene. **E**: Rescue of the *unc-68(r1161)* FLP-evoked reversal defect with the constructs shown in **D.** Bars indicate averages (+/- SEM). *n*≥80 animals. *, *p* < .01 versus *unc-68(r1161)* control by Dunnett’s tests. For each genotype, data are pooled from at least two independent transgenic lines showing similar results. **F**: Schematic of the *unc-68* constructs used to downregulate *unc-68* expression in specific tissues by RNA*i*. **G**: FLP-evoked reversal analysis showing the impact of tissue-specific *unc-6*8 RNA*i*. Bars indicate averages (+/- SEM). *n*≥80 animals. *, *p* < .01 versus wild type and respective sense controls by Bonferroni post-hoc tests. For each genotype, data are pooled from at least two independent transgenic lines showing similar results.

First, we determined whether the defect in *unc-68* null mutants could be rescued by tissue-specific expression of wild type UNC-68. We examined the effect of muscle-specific and pan-neuronal rescue by using previously generated transgenic lines, in which the large *unc-68* gene region contained in a cosmid had been recombined *in vivo* to be controlled by *myo-3* or *rab-3* promoters, respectively ([47], schematically depicted in Fig 8A). Whereas both transgenes markedly rescued swimming defects caused by the *unc-68(r1161)* mutation (Fig 8B), none of them rescued FLP-evoked reversals (Fig 8C). In order to interpret the absence of rescue with the *[rab-3p*::*unc-68]* pan-neuronal transgene, we next determined whether this version of the *rab-3* promoter was actually expressed in FLP. We generated transgenic animals containing a *[rab-3p*::*NLS*::*wrmScarlet]* construct to label in red the nuclei of cells where the *rab-3* promoter is active, and a *[mec-3p*::*CMK-1*::*mNeonGreen]* to locate FLP in green. The *rab-3* transcriptional reporter produced a strong signal in a large number of neurons throughout the animals, in the head, in the tail, as well as in motor neurons along the body. However, the expression in FLP was sporadic and extremely reduced as compared to that in motor neurons (S4 Fig). Together, these results suggest that the expression of *unc-68* in muscle or in a large number of neurons is not sufficient to maintain FLP-evoked reversals, but leave open the possibility that *unc-68* may directly act in FLP.

Next, we created cell-specific rescue transgenes for *unc-68*, targeting FLP more selectively. By using fosmid recombineering, we created a *[QUAS*::*unc-68]* rescue fosmid in which the endogenous *unc-68* promoter was replaced with the QUAS promoter. This promoter can be activated by the QF exogenous transcription factor under the control of a second promoter, in a two-component system ([48], see Fig 8D). We first verified that the *[QUAS*::*unc-68]* fosmid was functional by combining it with a *[unc-68p*::*QF*::*SL2*::*mCherry]* plasmid, which drives expression in all *unc-68*-expressing tissues. We observed a partial, but significant rescue of the FLP-evoked reversal defect in *unc-68(r1161)* null animals (Fig 8E). A control construct combination including a *[QUAS*::*unc-68(frameshift)]* fosmid did not produced any rescue effect. With this functional rescue system, we next combined the *[QUAS*::*unc-68]* fosmid with a *[mec-3p*::*QF*::*SL2*::*mCherry]* plasmid that drives robust expression in FLP and in the tail PLM neurons, and more sporadic expression in a few other neurons. This construct combination produced a partial rescue of FLP-evoked reversals, similar to that obtained with the *unc-68* promoter (Fig 8E). The partial rescue effect, observed even with the *unc-68* promoter, could be due to the mosaicism in extrachromosomal array-containing transgenic animals and/or to an inappropriate expression level with the two-component system. Although these data do not exclude the participation of additional neurons, they suggest that *unc-68* acts cell-autonomously into FLP to control stimuli-evoked reversals.

Third, we examined if a cell-specific knock-down of *unc-68* could be sufficient to impair FLP-evoked avoidance. To this end, we performed RNAi experiments by expressing complementary sense and antisense *unc-68* RNA sequences (Fig 8F). Inducing *unc-68* RNA*i* using the endogenous *unc-68* promoter successfully reduced FLP-evoked avoidance (Fig 8G). This effect was not as strong as that in *unc-68(r1161)* null, suggesting a partial loss of function with RNA*i*. No effect was found in controls where only the sense RNA was expressed. We obtained similar results when inducing RNA*i* with the *mec-3* promoter. Although the implication of other neurons is not excluded, our results suggest that UNC-68 expression in FLP is required for FLP-evoked avoidance.

### Deregulated UNC-68 function impairs FLP calcium response dynamic range

**Fig 9:**
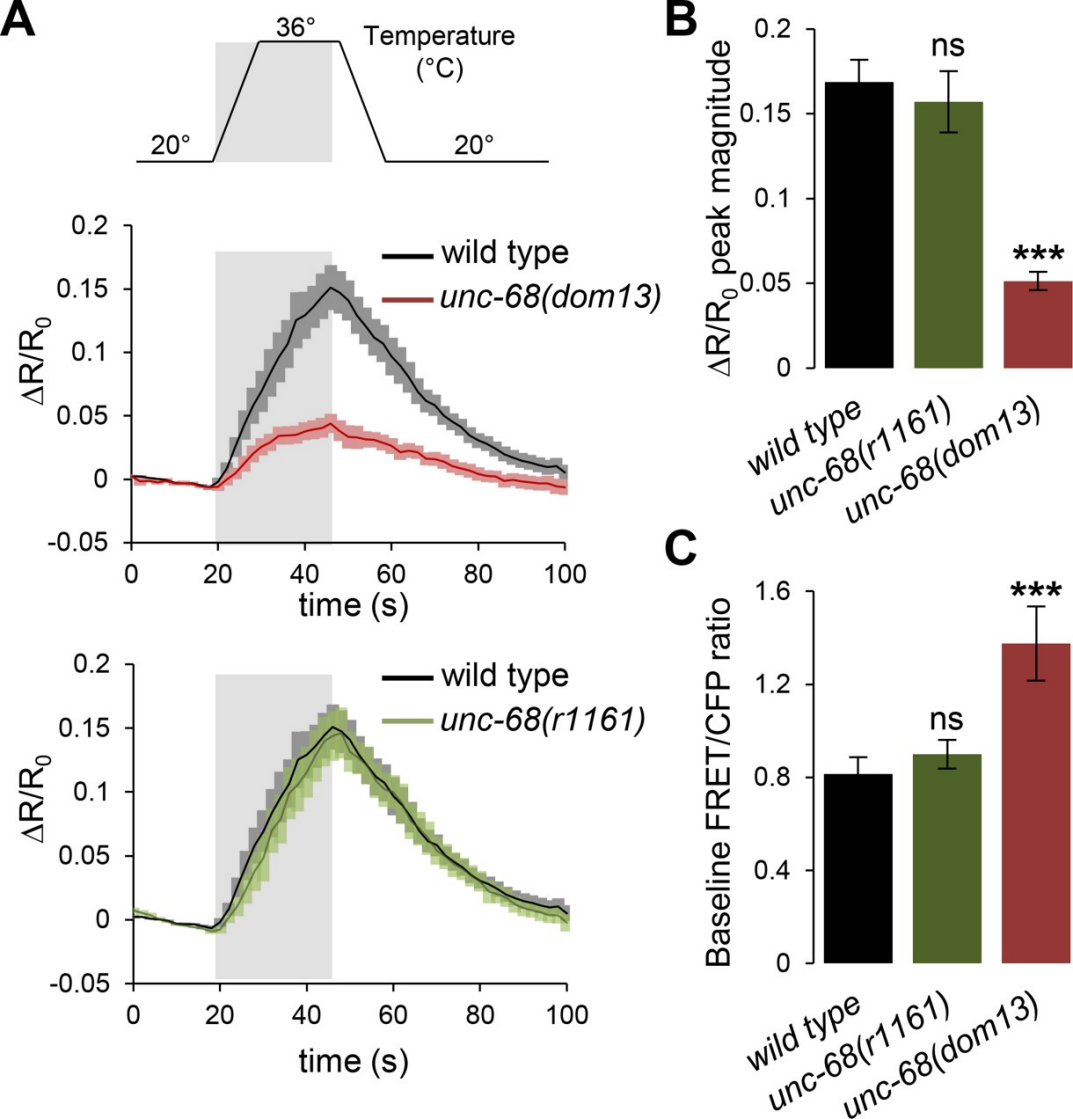
Impact of *unc-68* mutations on heat-evoked FLP calcium responses. **A**: FLP cell body calcium responses to 30 s noxious heat stimuli (top), measured in *[egl-46p*::*YC2*.*3]* transgenic animals expressing the YC2.3 Cameleon sensor. Solid lines indicate ΔR/R_0_ averages, with SEM as colored shades. **B**: Response peak comparison from data in **A** showing intact response in *unc-68(r1161)* loss-of-function mutants, but reduced response in *unc-68(dom13)* gain-of-function. Bars indicate average, with SEM as error bars. *n*≥11 recordings, each in separate animals. ***, *p <* .01 vs wild type by Dunnett’s tests; ns, not significant. **C**: Baseline FRET/CFP ratio (R_0_) at 20°C from the same recordings as in **A** and **B**, as a relative measure of the resting intracellular calcium level across animals of different genotypes. Bars indicate averages, with SEM as error bars. *n*≥11 recordings, each in separate animals. ***, *p <* .01 vs wild type by Dunnett’s tests; ns, not significant.

Since UNC-68 functions in FLP but does not appear to affect its development (Fig 6), we then evaluated whether it regulates the function of FLP. We tested the impact of *unc-68* mutations on the FLP response to noxious heat stimuli, in a line expressing a Cameleon calcium indicator in FLP [11]. Wild type animals maintained at 20°C produced robust calcium peaks in FLP cell bodies in response to a 36°C heat stimulation, as evidenced with a marked change in the baseline-normalized relative change in FRET/CFP ratio (ΔR/R_0_)(Fig 9A). This response was not significantly affected in *unc-68(r1161)* mutants, but was significantly reduced in *unc-68(dom13)* mutants by a factor of two (Fig 9A and 9B). A decreased ΔR/R_0_ change may reflect a decreased responsiveness to heat, or be due to a celling effect if baseline cytosolic calcium is increased in the mutant. To examine this possibility, we verified that the Cameleon expression level was similar across genotypes (*p*>.41 by Bonferroni contrasts) and then analyzed the raw FRET/CFP ratio at 20°C (baseline) across animals (Fig 9C). Baseline FRET/CFP ratios, which reflect the absolute calcium levels at rest, were identical between wild type and *unc-68(r1161)* mutants, but significantly increased in *unc-68(dom13)* mutants. These results show that the impairment in noxious heat-evoked responses observed in *unc-68(dom13)* reflects a reduced calcium response dynamic range, potentially through a celling effect linked to elevated baseline calcium levels in non-stimulated conditions.

## Discussion

### Forward optogenetics

By allowing the selective activation of specific neurons with a high spatiotemporal accuracy, optogenetics has revolutionized the investigations of the functions of specific neurons within their respective circuits [49]. One of the current challenges is to comprehensively investigate the molecular mechanisms that control the many neural pathways composing these circuits. This requires first to identify functionally relevant molecular players, despite the overall system robustness that is usually conferred by the functional architecture of these circuits. Previous studies have successfully used optogenetic stimulations to screen for genes controlling neuronal functions [50],[51]. In these cases, screens were performed using RNA*i* from a pre-selected list of candidates (<100 candidates), defined from separate gene expression analyses. We present here an efficient and versatile pipeline combining optogenetic stimulations with the power of ‘classical’ mutagenesis screen and whole genome sequencing-based mutation mapping with VDM. The main advantages of our approach are that it (i) allows a genome-wide interrogation, (ii) does not require prior information, (iii) examines the impact of both loss and gain of functions, and (iv) can identify genes whose alterations produce less widespread impacts in the nervous system, but which are nevertheless crucial for specific types of neurons.

To demonstrate the utility of our strategy, we addressed the function of FLP, a polymodal mechano/thermosensory nociceptive neuron, whose sensory functions are ‘backed-up’ by several parallel sensory pathways. According to our expectations, screening mutants for defects in reversals induced by FLP optogenetic activation allowed the recovery of mutations in both ‘general’ genes mediating aversive behavior, and genes with a narrower implication, notably into the FLP-dependent pathway.

### ‘General’ avoidance genes

We recovered three avoidance gene mutations producing a general impact on reversal behavior induced by a wide range of stimuli: *unc-4*, *eat-4* and *unc-83*. Loss of *unc-4* function was previously shown to misconnect the AVA command neurons toward B motor neurons driving forward locomotion, rather than toward A motor neurons driving backward locomotion [25, 52]. As AVA is postsynaptic to FLP, this miswiring is the most likely explanation for the mutant phenotypes observed in the present study. *unc-4* represents an example of a ‘general’ avoidance gene, whose ‘universal’ requirement is due to its function in a central interneuron (AVA), on which multiple sensory pathways converge (S1 Fig).

The second ‘general’ avoidance gene, *eat-4*, plays an essential role for glutamatergic neurotransmission. In fact, we used a previously identified allele of *eat-4* as positive control when establishing the screen conditions and we were expecting to recover additional loss-of-function alleles during our screen. Its broad phenotypic impact is not surprising, as most of the behavioral assays carried out in the present study actually involve glutamatergic sensory neurons (AFD, ALM, ASH, AVM, AWB, FLP, OLQ). *eat-4* represents therefore an example of a ‘general’ avoidance gene, whose widespread role is explained by its broad expression across several sensory neurons.

The third ‘general’ avoidance gene is *unc-83*, which encodes a KASH protein [30] required for nuclear migration, particularly within syncytial cells [31]. Loss of *unc-83* affects animal development, notably causing defects in the ventral cord neurons [32, 33]. We found no obvious developmental defect in FLP in *unc-83(dom12)* mutants. The simplest explanation for the reversal response defects in *unc-83* mutants would be that they are caused by developmental abnormalities of the motor neurons mediating the execution of backward locomotion. More investigations will be required to elucidate the role of *unc-83* and of its multiple isoforms in controlling avoidance behavior.

### ‘FLP-pathway specific’ avoidance genes

In addition to avoidance genes with widespread action, our screen also identified three genes whose mutation more selectively impaired FLP-dependent behaviors: *syd-2*, *unc-14* and *unc-68*. To our knowledge, our study is the first to show a role in nociceptive pathways for these genes, highlighting the interest of neural pathway-specific genetic screen using optogenetics. Our approach can thus complement past genetic analyses with natural stimuli and highlight novel molecular pathways.

Both *unc-14* and *syd-2* are broadly expressed in the nervous system and play a role in the formation of functional synaptic connections. UNC-14 is involved in axonogenesis [53], axon elongation and guidance [27] and in the regulation of synaptic vesicle localization. SYD-2 is involved in synaptogenesis, size and stability of pre-synaptic structures [34, 36, 37, 54] and regulation of dense core vesicle polarized trafficking [35]. Our data suggest that FLP-evoked behaviors involve the expression of *unc-14* and *syd-2* in FLP and potentially in other neurons, which could be downstream neurons in the FLP-pathway. Additional studies will be required to determine how *syd-2* and *unc-14* control aversive behaviors and why their function seems to be selectively required for the response to specific noxious stimuli through specific neural pathways, in spite of their relatively broad expression pattern.

### The *C*. *elegans* Ryanodine receptor UNC-68 controls nociception

UNC-68 is the only orthologue of mammalian Ryr1, Ryr2, and Ryr3 channel subunits. In the nervous system, UNC-68 plays a role in synaptic transmission by motor neurons [47], in the regulation of axon degeneration and regeneration in mechanosensory neurons [55, 56] and in the tonic sensory signaling in oxygen-sensing neuron [57]. These UNC-68 neuronal functions determined so far were mostly deduced from the phenotype of *unc-68* null alleles. The UNC-68 G4996D mutation recovered in our screen is most likely a gain-of-function. This conclusion is supported by allele interaction analyses, phenotypic analyses on avoidance behavior, swimming and pharyngeal pumping, as well as the elevated level of cytoplasmic calcium measured in FLP. Up-regulation of Ryr3 was previously shown to increase resting levels of cytoplasmic calcium in mammalian cells [58]. The glycine 4996 of UNC-68 is conserved in all RyR isoforms from *C*. *elegans* to human (G4935 in Ryr1, G4864 in Ryr2 and G4767 in Ryr3 from human) and forms the so-called glycine hinge around which the S6 transmembrane kinks to take its final shape in the closed channel structure (see Fig 4A, [59]). Functional studies in mammalian Ryr2 examining the biophysical properties of mutant channels with proline, alanine and valine substitutions have indicated that this glycine is essential for normal opening or closing kinetics [60][61], and a similar mechanism could be involved in *unc-68(dom13)* mutants.

Our results with gain- and loss-of-function mutations indicate that properly regulated UNC-68 activity levels are required for normal nociceptive response in the FLP-pathway. While not excluding an action in other cells, we found that UNC-68 acts cell-autonomously in FLP to control reversal behaviors. In an attempt to understand how UNC-68 controls FLP sensory activity, we found that gain of UNC-68 function increased the baseline cytoplasmic calcium level in FLP cell bodies and reduced the magnitude of heat-evoked calcium peaks. This constitutes a potential mechanism through which UNC-68 could control FLP sensory signal transduction activity and could explain the marked defect in noxious heat evoked reversal response in *unc-68(dom13)* gain-of-function mutants. Loss of UNC-68 produced a less pronounced heat-evoked reversal defect, but no detectable calcium signaling alteration. It is possible that loss of UNC-68 affects heat-evoked reversals by more subtly modifying FLP function, e.g. by affecting more local calcium signals in neurites, as it does during axon regeneration [55], and/or by affecting additional thermosensory neurons. Collectively, the results of our screen follow up analyses on *unc-68* demonstrate the importance of properly regulated intracellular calcium store recruitment by Ryanodine receptors in the FLP pathway and illustrate the utility of our new “forward optogenetics” approach to identify relevant genetic entry points for functional analyses.

### Conclusion

In conclusion, our work represents a proof of-concept for a genome-wide forward optogenetic approach in *C*. *elegans*, which can be easily expanded to dissect any specific neural pathway where an optogenetic intervention triggers a detectable behavioral response. Of note, optogenetic activation does not need to be limited to sensory neurons and could be performed elsewhere in the circuit (e.g., in interneurons). In addition, a more comprehensive phenotype quantification could be applied using more elaborated computer-assisted behavioral analyses [62, 63]. Forward optogenetics could become a powerful mean to uncover gene functions that have not yet been discovered because of the intrinsic robustness of the nervous system functional architecture. Ultimately, this approach may provide a more complete view of the mechanisms regulating sensory behaviors and other nervous system functions.

## Methods

### *C*. *elegans* strains and growth conditions

The *C*. *elegans* strains used in this study are listed in S2 Table. All strains were grown at 20 or 23°C on nematode growth media (NGM) plates with OP50 *Escherichia coli* as previously described [64]. For optogenetic experiments, we prepared plates with or without ATR. When ATR was included, we added 0.1% (v/v) of ATR stock (100 mM, in ethanol) to the OP50 bacteria suspension, and seeded each NGM agar plate (60 mm diameter and 15 mm height, containing 8 ml NGM) with 300 μl of this mix. For mechanosensation behavioral assays, we made NGM plates with a thinner layer of freshly grown OP50 bacterial culture (50 μl).

### Mutagenesis screen protocol

We based our mutagenesis protocol on articles by Jorgensen and Mango [65] and by Shaham [66]. The screen was carried out in small batches, until reaching 2460 separate F1. DAG356 animals containing *domIs355[FLP*::*CoChR]* transgene were treated with 50mM ethyl metanesulphonate (EMS) mutagen for 4h in M9 solution in a 15 mL Falcon tube submitted to continuous rotation, as previously described [65]. Two hundred mutagenized worms (P0) were transferred into NGM plates, 10 worms per plate for a total of 20 plates and allowed to recover and self-fertilize. From each P0 plate, we picked 10 F1 animals into a fresh, ATR-containing plate and allowed egg laying. Then, the F2 generation was tested for light-evoked behaviors using a 5 trial stimulation protocol with 61 W/m^2^ blue light intensity (see screen conditions above). We scored twenty-five F2 worms in each plate, making an average of 2.5 F2 per F1 analyzed for light-induced behaviors. We recovered a maximum of one candidate mutant line for each plate, ensuring that each line represented an independent mutagenesis event. The candidate mutant lines with defective light-avoidance behavior were isolated to a fresh plate and at least two subsequent generations were reevaluated to verify if the phenotype was maintained. Mutant lines were backcrossed at least three times with the parental line DAG356 before mapping and further analyses.

### Mapping strategy, Genomic DNA preparation and Whole Genome Sequencing (WGS)

We followed the variant discovery mapping strategy described in [24]. We backcrossed each mutant strain retrieved from the screen with the parental optogenetic strain (DAG356) and picked F1 cross progeny. Then, from the F2 progeny, we isolated 50 worms with defective light-avoidance phenotype onto individual plates. This number was higher than previous recommendations in order to obtain a more restricted mapping region. We let the F2 recombinants to self-fertilize until plates were full of worms and starved for no more than one day. Then, we pooled them using a similar amount of worms from each of the 50 plates. We extracted the worms’ genomic DNA according to a standard protocol [67]. DNA libraries were prepared according to Illumina’s protocol. The raw reads (paired-end 150bp HiSeq3000) were obtained from the NGS platform of the University of Bern.

### Mutation mapping

For *dom8*, *9*, *11*, *12 and* 13, the raw reads were used as an input to the previously described Variant Discovery Mapping CloudMap pipeline[68], running on an in-house Galaxy server. Variants present in the parental/backcrossing strain DAG356 (which was also whole genome sequenced) were subtracted from each mutant strain when determining the list of potential causal variants.

For *dom15*, we run a similar analysis protocol with the following step sequence. After quality check with FastQC (https://www.bioinformatics.babraham.ac.uk/projects/fastqc/), all the reads where filtered for quality with Trimmomatic[69] (LEADING:10 TRAILING:10 SLIDINGWINDOW:4:15 MINLEN:130) and then mapped with bwa mem v0.7.13[70] to the *C*. *elegans* reference genome obtained from ENSEMBL[71]. The sam file were sorted and converted to bam with Samtools v1.3[72]. The duplicates were marked with Picard-tools (http://broadinstitute.github.io/picard/). The reads were realigned around indels and the base score recalibrated. The variants were called with the Haplotype caller leading to gvcf files that were jointly merged with GenotypeGVCFs to the unannotated vcf file using GATK recommended pipeline [73, 74]. The annotation was added with SnpEff v4.3q[75] and the variants filtered with SnpSift[76] to keep variants with HIGH and MODERATE impact that are not found in the parental line leading to an annotated and curated vcf file.

### Behavioral assays

All experiments were performed blindly with respect to the genotypes. All experimental replicates were obtained over at least 3 independent days and unless otherwise indicated, adult worms were used either synchronized by bleaching or picked as L4 larvae on NGM plates one day before the experiments.

#### Response to FLP and ASH optogenetic activation

Single forward moving animals were illuminated with 61 W/m^2^ blue light during 0.5 s for FLP and with 202 W/m^2^ for 3 s for ASH. Scoring was done manually and any backward movement taking place during the stimulation was counted as a positive response. Animals were stimulated five times in a row. Each animal received a response score over these five trials. Scores were then averaged over the animals for each genotype.

#### Harsh touch assays

Harsh touch was delivered with a platinum wire pick as previously described [77]. The stimulus was applied from above the animals by pressing down with the edge of the pick with an estimated force of 100 μN on the head of a forward moving adult animal. We scored as positive responses any reversal or stop. To avoid any confounding effect of physical damage, each animal was tested only once. Results are presented as fractions of animals that were responding.

#### Noxious heat assays

Noxious heat stimuli were delivered to 6-cm NGM plates containing 50–150 animals with four 100 W infra-red lamps heating the plate at a rate of ~2°C/s during 4 seconds. Baseline temperature was 20°C, maintained by a Peltier-cooled aluminum plate below the NGM plate. We recorded videos with a DMK23UP1300 camera (The Imaging Source, Germany) and manually scored the heat-evoked responses over 10 trials with an interstimulus interval of 20 seconds. Any backward movement observed during the 4 s stimulation was counted as a positive response. Each animal received a response score over these ten trials. Scores were then averaged over the animals for each genotype.

#### Nose touch assays

Nose touch was tested by laying an eyelash hair on the surface of the plate perpendicularly to the worms’ forward movement and let them bump into it, as previously described [78]. We scored as a positive response either a reversal or a stop. Each worm was tested 10 times in a row. Each animal received a response score over these ten trials. Scores were then averaged over the animals for each genotype.

#### Anterior gentle touch assays

Anterior gentle touch stimuli were delivered with an eyelash by touching the animals just behind the pharynx. We scored as positive responses reversals and stops. To allow comparison with harsh head touch assays, a single trial was used. Results are presented as fractions of animals that were responding.

#### 2-nonanone avoidance assays

Animals starved for 2 hours were tested on food-free NGM plates. Videos of worm behavior were recorded during which a 2 μl drop of 2-nonanone (Sigma, Switzerland) was deposited in one corner of the field of view. Animals that were within 10 mm of the drop but not in direct contact with it were scored for reversal. Results are presented as fractions of animals that were responding with a reversal.

#### Pharyngeal pumping assays

A stereomicroscope (Leica M2015FA) equipped with a camera (Leica DFC345FX) was used at a 160X magnification to record videos of grinder movements of adult animals on food. Grinder movements were scored manually over 20 s to determine pumping rate and inter-beat intervals. Each video was analyzed twice and results, which only negligibly diverged, were averaged.

### Microscopy and transcriptional reporter activity measurements

To assess FLP cell body localization and to quantify *mec-3p* and *rab-3p* reporter activity, we used an Axio Plan 2 fluorescence microscope (Zeiss) with a 40x objective (air, NA = 0.95) and constant illumination parameters. At least 40 animals per genotype were analyzed using Fiji/ImageJ. For *rab-3p* expression analysis in motor neurons, we quantified the signal from the first visible motor neuron directly anterior to the vulva (presumably VC4).

To qualitatively assess FLP arborization in wild type and mutants, we used a Leica TCS SPE-II confocal microscope (APO 40x oil objective, NA1.15), equipped with a 488nm wavelength diode laser and a ET525/50m emission filter. Z-stack images were acquired across whole animal thickness. Maximal intensity projections are depicted in the figures [79].

### Calcium imaging

#### Animal preparation

0.3 mm-thick agarose pads were prepared with 2% agarose in M9 buffer (KH_2_PO_4_ 22 mM, Na_2_HPO_4_ 22 mM, NaCl 85 mM, MgSO_4_ 1 mM) using glass coverslips as support, as previously described[80]. Transgenic *ljEx19[pegl-46*::*YC2*.*3 lin-15(+)]* animals expressing the Cameleon sensor in their FLP neurons were pre-selected by using an epi-fluorescence stereomicroscope at least one hour prior to the experiment. For each recording, one worm was collected with an eyelash pick, transferred to an agarose pad, and then immobilized by applying Dermabond surgical glue (Closure Medical), using glass capillary needles operated by mouth. The glue was initially applied close to the tail of the worm, on its dorsal side (keeping the ventral side free of glue in order to allow intact egg laying physiology) and then along the whole body, until close to the head but without gluing the nose. After glue polymerization (around 30 s), M9 buffer was added close to the worm to keep the environment slightly humid and prevent exsiccation of the agarose pads. A plastic spacer was placed on the coverslip between the worm and a Cherry Temp microfluidic chip (Cherry Biotech, Rennes, France). Sample temperature could be stably maintained (variations < 0.1°C) or quickly changed to a new steady temperature (within 10 s), thanks to a circuit switch between two pre-equilibrated Peltier elements located near the sample. The temperature of the microfluidic chamber was preset to 20°C to maintain a constant baseline temperature before initializing the experimental protocols. Stimulation was at 36°C for 30 s.

#### FRET imaging

Worms were imaged using an inverted epifluorescence microscope (Leica DMI6000B) with aHCX PL Fluotar L40x/0.60 CORR dry objective, a Leica DFC360FX CCD camera (1.4M pixels, 20 fps), an EL6000 Light Source, and fast filter wheels for FRET imaging. Excitation filters for CFP and FRET: 427 nm (BP 427/10). Emission filters for CFP: 472 (BP 472/30) and for FRET: 542 nm (BP 542/27). Dichroic mirror: RCY 440/520. Five minutes of initial recording at 20°C was used as baseline. The acquisition rate was 1Hz.

#### Signal analyses

All time-lapse recordings were analyzed using LAS X software (Leica). To calculate FRET ratio, we selected a first region of interest (ROI), delimiting the background, and a second ROI, which included FLP soma. For each channel, the ROI _background_ values were subtracted from the ROI_FLP_ values. The resulting values for FRET and CFP respectively (namely FRET_FLP-background_ and CFP_FLP-background_) were divided and the FRET ratio (FRET/CFP) was calculated as follows:

$$R=FRET\;ratio=\frac{{FRET}_{FLP-background}}{{CFP}_{FLP-background}}$$

To express the relative change in intracellular calcium levels (Fig 9A and 9B), the FRET ratio *R* was normalized and expressed as a Δ*R/R*_*0*_ at every time point, where Δ*R* is *R*-*R*_*0*_ and *R*_*0*_ is the average of *R* over the last 30 s prior to stimulus onset. To compare baseline intracellular calcium levels between genotypes, we first verified that the Cameleon sensor was expressed at similar levels between them. To do so, we computed a total fluorescence index (FRET_FLP-background_ + CFP_FLP-background_) as an indicator of the Cameleon sensor expression level, and found it was not significantly different between genotypes (all *p*>.41 by Bonferroni contrasts). Then, we compared the baseline FRET ratio *R_0_* between genotypes (as reported in Fig 9C).

### Transgenesis

DNA prepared with a GenElute HP Plasmid miniprep kit (Sigma) was microinjected in the gonad to generate transgenic lines according to a standard protocol [81]. We used a *[unc-122p*::*GFP]* co-injection marker to identify transgenic animals [18].

### Cosmids and fosmids

F23K23 *[eat-4 fosmid]*, M04C11 *[unc-68 cosmid]*, and W01A11 *[unc-83 cosmid]* were obtained from the Wellcome Sanger Institute (Hinxton Cambridge, UK).

WRM069cA02 *[unc-68 fosmid]* was obtained from Source BioScience. The generation of the *[QUAS*::*unc-68]* fosmids is described below.

### Promoter plasmids (MultiSiteGateway slot 1)

Entry plasmids containing specific promoters were constructed by PCR from N2 genomic DNA with primers flanked with attB4 and attB1r recombination sites and cloned into pDONR-P4-P1R vector (Invitrogen) by BP recombination. Primer sequences were the following:

**dg596** *[slot1 Entry unc-14p]*

attB4unc-14_F: ggggacaactttgtatagaaaagttgatcaaagagagaggtggaaactgaaca

attB1runc-14_R: ggggactgcttttttgtacaaacttgtttgagggatgaagccctttgtag

**dg598** *[slot1 Entry syd-2p]*

attB4syd-2_F: ggggacaactttgtatagaaaagttgatagtgagtttgactggctctact

attB1rsyd-2_R: ggggactgcttttttgtacaaacttgtaccttgcagaaagtgaataacctaca

**dg600** *[slot1 Entry unc-4p]*

attB4unc-4_F: ggggacaactttgtatagaaaagttgattatgaatgctgtcccggaactg

attB1runc-4_R: ggggactgcttttttgtacaaacttgttttcactttttggaagaagaagatcctct

**dg712** *[slot1 Entry eat-4p]*

attB4eat-4_F: ggggacaactttgtatagaaaagttgatgtgccatccgtctatttccagaatga

attB1reat-4_R: ggggactgcttttttgtacaaacttgtctgaaaatgatgatgatgatgatggagttg

**dg604** *[slot1 Entry unc-68p]*

attB4unc-68AB_F: ggggacaactttgtatagaaaagttgatcgttggttaataattgttggctaaccgt

attB1runc-68AB_R: ggggactgcttttttgtacaaacttgtctgtaaaacaaaaaaactagaggtgctgg

**dg672** *[slot1 Entry rab-3p1208]*

attB4rab-3p1208_F: ggggacaactttgtatagaaaagttgatgatcttcagatgggagcagtggact

attB1rrab-3p1208_R: ggggactgcttttttgtacaaacttgtagccgccatctgaaaatagggcta

The generation of **dg68** *[slot1 Entry mec-3p_noATG]* and **dg229** *[slot1 Entry QUASprom]* was previously described [19].

### Coding sequence plasmids (MultiSiteGateway slot 2)

Entry plasmids containing specific coding DNA sequences (cds) were constructed by PCR from N2 cDNA with primers flanked with attB1 and attB2 recombination sites and cloned into pDONR_221 vector (Invitrogen) by BP recombination. Primer sequences were the following:

**dg595** *[slot2 Entry unc-14cds]*

attB1unc-14_F: ggggacaagtttgtacaaaaaagcaggcttaatggtggaactgtgcgaactgca

attB2unc-14_R: ggggaccactttgtacaagaaagctgggttcaaactcggttgaattgggtggcat

**dg597** *[slot2 Entry syd-2cds]*

attB1syd-2_F: ggggacaagtttgtacaaaaaagcaggcttaatgagctacagcaatggaaacataa

attB2syd-2_R: ggggaccactttgtacaagaaagctgggtttcgtttagactagcacgaatga

**dg599** *[slot2 Entry unc-4cds]*

attB1unc-4_F: ggggacaagtttgtacaaaaaagcaggcttaatgatcggtgcactgcatgcat

attB2unc-4_R: ggggaccactttgtacaagaaagctgggttaaataaccaatggagggcacggttc

**dg713** *[slot2 Entry eat-4cds]*

attB1eat-4_F: ggggacaagtttgtacaaaaaagcaggcttaatgtcgtcatggaacgaggct

attB2eat-4_R: ggggaccactttgtacaagaaagctgggttgtagttctggtttttcgcaactaggc

**dg621** *[slot2 Entry unc-68 RNAi sense]*

attB1unc-68RNAis_F: ggggacaagtttgtacaaaaaagcaggcttactgagagcagttcgattaggtccaa

attB2unc-68RNAis_R: ggggaccactttgtacaagaaagctgggttctgaaggacctccttcggaaattgt

**dg622** *[slot2 Entry unc-68 RNAi antisense]*

attB1unc-68RNAias_F: ggggacaagtttgtacaaaaaagcaggcttatctgaaggacctccttcggaaattgt

attB2unc-68RNAias_R: ggggaccactttgtacaagaaagctgggttctgagagcagttcgattaggtccaa

The generation of **dg240** *[slot2 Entry QF_withATG]* was previously described [19].

To create **dg651** *[slot2 Entry egl-13NLS*::*wrmScarlet]*, we first obtained a codon-optimized *wrmScarlet* version containing three artificial introns via gene synthesis (Eurofins DNA), amplified it by PCR to subclone it into pDON221 by BP recombination (creating dg642), and added the *egl-13NLS* by PCR. We used the following primers:

NLSRightHalf_wrmScarlet_F: AAAACGCGAAGAAGCTTGCCAAGGAAGTTGAAAATGGATCCATGGTCAGCAAGG

NLSLeftHalf_Entry_R: CACTCAGTTTTGTCGGATTCGCTTTTCGTCTACGGCTCATGTTGCTAGCGGTACCTAAG

### 3’ UTR and tagging plasmids (Multi-site Gateway slot3)

**mg277** *[SL2*::*mCherry]* was previously described [18].

**mg211** *[EntrySlot3unc-54UTR]* (aka pMH473) was a gift from Marc Hammarlund.

### Selection marker used for transgenesis

**dg396** *[coel*::*GFP]* (or *unc-122p*:*GFP*) was a gift from Piali Sengupta (Addgene plasmid # 8937)

### Expression plasmids used for transgenesis

**dg243** *[mec-3p*::*QF*::*unc-54UTR]* was previously created through a LR recombination reaction between dg68, dg240, mg211, and pDEST-R4-P3.

**dg571** *[unc-4p*::*unc-4cds*::*SL2*::*mCherry]* was created through a LR recombination reaction between dg600, dg599, mg277, and pDEST-R4-P3.

**dg715** *[eat-4p*::*eat-4cds*::*SL2*::*mCherry]* was created through a LR recombination reaction between dg712, dg713, mg277, and pDEST-R4-P3.

**dg572** *[unc-14p*::*unc-14cds*::*SL2*::*mCherry]* was created through a LR recombination reaction between dg596, dg595, mg277, and pDEST-R4-P3.

**dg574** *[syd-2p*::*syd-2cds*::*SL2*::*mCherry]* was created through a LR recombination reaction between dg598, dg597, mg277, and pDEST-R4-P3.

**dg717** *[mec-3p*::*unc-4cds*::*SL2*::*mCherry]* was created through a LR recombination reaction between dg68, dg599, mg277, and pDEST-R4-P3.

**dg716** *[mec-3p*::*eat-4cds*::*SL2*::*mCherry]* was created through a LR recombination reaction between dg68, dg713, mg277, and pDEST-R4-P3.

**dg709** *[mec-3p*::*unc-14cds*::*SL2*::*mCherry]* was created through a LR recombination reaction between dg68, dg595, mg277, and pDEST-R4-P3.

**dg710** *[mec-3p*::*syd-2cds*::*SL2*::*mCherry]* was created through a LR recombination reaction between dg68, dg597, mg277, and pDEST-R4-P3.

**dg628** *[mec-3p*::*unc-68senseRNAi*::*SL2*::*mCherry]* was created through a LR recombination reaction between dg68, dg621, mg277, and pDEST-R4-P3.

**dg629** *[unc-68pAB*::*unc-68senseRNAi*::*SL2*::*mCherry]* was created through a LR recombination reaction between dg604, dg621, mg277, and pDEST-R4-P3.

**dg632** *[mec-3p*::*unc-68antisenseRNAi*::*SL2*::*mCherry]* was created through a LR recombination reaction between dg68, dg622, mg277, and pDEST-R4-P3.

**dg634** *[unc-68p*::*unc-68antisenseRNAi*::*SL2*::*mCherry]* was created through a LR recombination reaction between dg604, dg622, mg277 and pDEST-R4-P3.

**dg648** *[unc-68pAB*::*QF*::*unc-54UTR]* was created through a LR recombination reaction between dg604, dg240, mg211, and pDEST-R4-P3.

**dg673** *[rab-3p1208*::*egl-13NLS*::*wrmScarlet*::*unc-54UTR]* was created through a LR recombination reaction between dg672, dg651, mg211, and pDEST-R4-P3.

### Generation of *[QUAS*::*unc-68]* rescue fosmids

**dg639** *[QUAS*::*unc-68(wt)]* and **dg631** *[QUAS*::*unc-68(fs)*, *frame shift]* rescue fosmids were obtained by fosmid recombineering, using the screening strategy of kanamycin resistance gene (kanR) insertion as previously described [82]. We first obtained via gene synthesis a plasmid containing a *[KaQU68(fs)]* construct fusing: (i) a 100 base homology stretch of the pCC1FOS fosmid backbone, (ii) *kanR*, (iii) the QUAS promoter sequence and (iv) the first one hundred nucleotides of the *unc-68* gene (with a frameshift in exon1). A *[KaQU68(wt)]* version was then obtained by site directed mutagenesis. Then we amplified each construct by PCR with the following primers:

KaQU68_F1: cacaggaaacagctatgaccatgatt
KaQU68_R1: gtcggccatcgttgtcgat

Resulting products were then recombined *in vivo* in *E*. *coli* with the *unc-68* containing WRM069cA02 fosmid, according to the protocol developed by Tursun and collaborators [82]. Briefly, we first electroporated the WRM069cA02 fosmid into SW105 *E*. *coli* cells, followed by induction of the λ red recombinase and preparation electro-competent λ red-activated SW105 bacteria. Each of the PCR products described above were then separately electroporated into batches of these cells, which were then plated on kanamycin plates and let to grow 36h at 32°C. Finally, we identified the grown colonies carrying the recombineered fosmid by PCRs using the following primers:

pCC1FOS_F1: cccaggctttacactttatgcttc
unc68_exon1R: gagaaaagagacatcatcctgttcg
unc68_promR1: tccttctatcaaacttacgtggctt

Fosmids from colonies generating the expected band patterns were electroporated back into the bacterial strain EPI300 for storage and maintenance.

## Supporting information

S1 Fig Anterior thermosensory and mechanosensory neurons and core circuit mediating backward locomotion. The scheme was inspired by Goodman, 2006 [15].
(PDF)

S2 Fig Variant Discovery Mapping plots. **(A, B, C, D, E)** The allele frequencies of high-quality variants (ratio of variants reads/total reads) identified in whole-genome sequenced pools of homozygous mutant recombinants are plotted as a function of genomic coordinates. The green line is a LOESS (locally estimated scatterplot smoothing) local regression to help identify the linked (peak) region. Protein coding mutations located in the peak region are indicated with rectangular labels (red for the causal mutation and yellow for other linked non-causal mutations).
(PDF)

S3 Fig Overview of behavioral impairments in mutants. Reversal analysis results from Figs 3, 4 and 5 pooled in a single heat map. Data are expressed according to the depicted color scale as a fraction of wild type responsiveness.
(PDF)

S4 Fig The *rab-3* promoter used for ‘pan-neuronal’ unc-68 rescue drives nearly undetectable expression in FLP. **A**: Schematic of the three transgenes used and their roles. **B**, **C**: Representative epifluorescence micrographs from a single animal revealing both green and red signals, using a high pass emission filter and a color camera to visualize both channels. Only green signal is detectable in FLP (**B**, top inset), while red nuclei are detected in PLM (**C**, lower left inset) and a large number of other neurons (including motor neurons, **C**, lower right inset). **D**: Quantification of [rab-3p::wrmScarlet] signal in FLP and motor neurons. FLP nuclei were localized thanks to the mNeonGreen signal. Acquisition for quantification was made with a RFP filter cube to selectively quantify the wrmScarlet signal. Note the interrupted vertical axis. Grey bars indicate averages, with SEM as error bars. *, *p* < .001 by Student’s *t*-test. Data are pooled from two independent transgenic lines showing similar results.
(PDF)

S1 Table New alleles recovered in the genetic screen. (PDF)

S2 Table Strains used in this study. (PDF)

S1 File Numerical data underlying summary statistics. (XLSX)
