# Supplementary material for: Identification of avoidance genes through neural pathway-specific forward optogenetics
Source: PLoS Genet. 2019 Dec 31;15(12):e1008509. doi: 10.1371/journal.pgen.1008509 (PMC6938339; doi:10.1371/journal.pgen.1008509)
Supplement: S1 Fig — The scheme was inspired by Goodman, 2006 [15]. (PDF) [file pgen.1008509.s001.pdf]

Figure S1

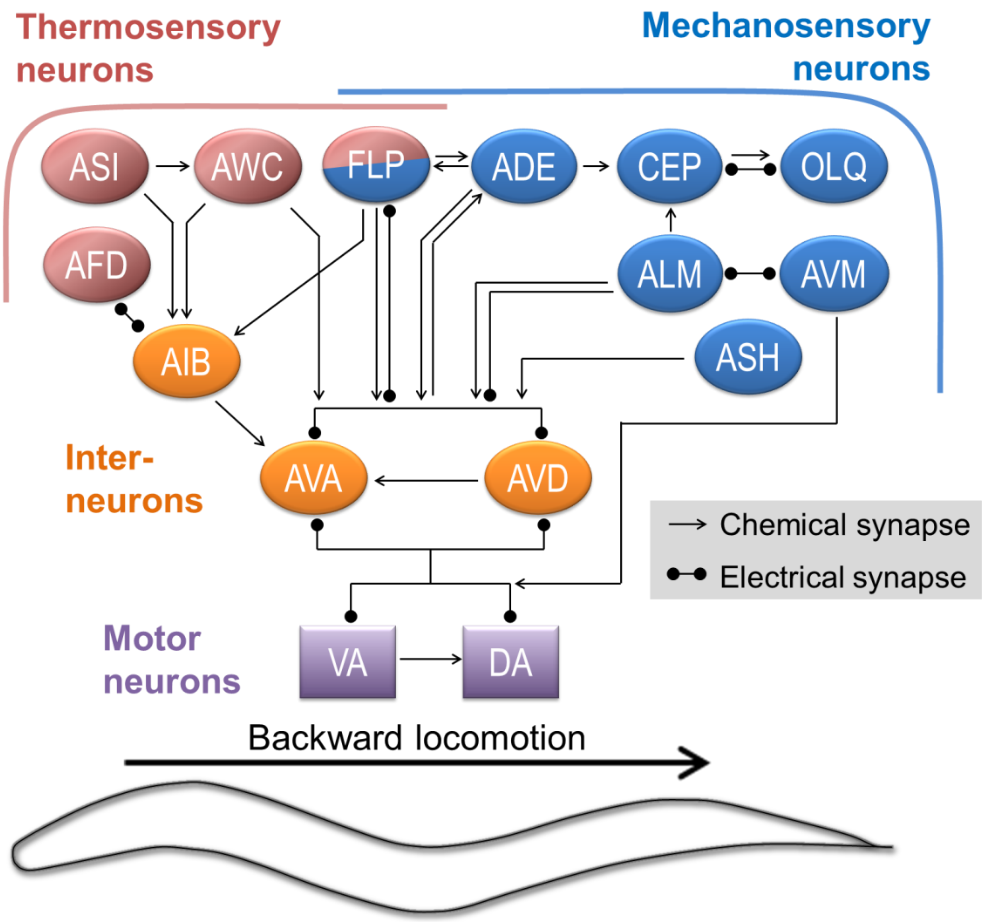

Figure S1: Anterior thermosensory and mechanosensory neurons and core circuit mediating backward locomotion. The scheme was inspired by Goodman 2006 [15].
