# Supplementary material for: Identification of avoidance genes through neural pathway-specific forward optogenetics
Source: PLoS Genet. 2019 Dec 31;15(12):e1008509. doi: 10.1371/journal.pgen.1008509 (PMC6938339; doi:10.1371/journal.pgen.1008509)
Supplement: S2 Fig — (A, B, C, D, E) The allele frequencies of high-quality variants (ratio of variants reads/total reads) identified in whole-genome sequenced pools of homozygous mutant recombinants are plotted as a function of genomic coordinates. The green line is a LOESS (locally estimated scatterplot smoothing) local regression to help identify the linked (peak) region. Protein coding mutations located in the peak region are indicated with rectangular labels (red for the causal mutation and yellow for other linked non-causal mutations). (PDF) [file pgen.1008509.s002.pdf]

S2 Figure

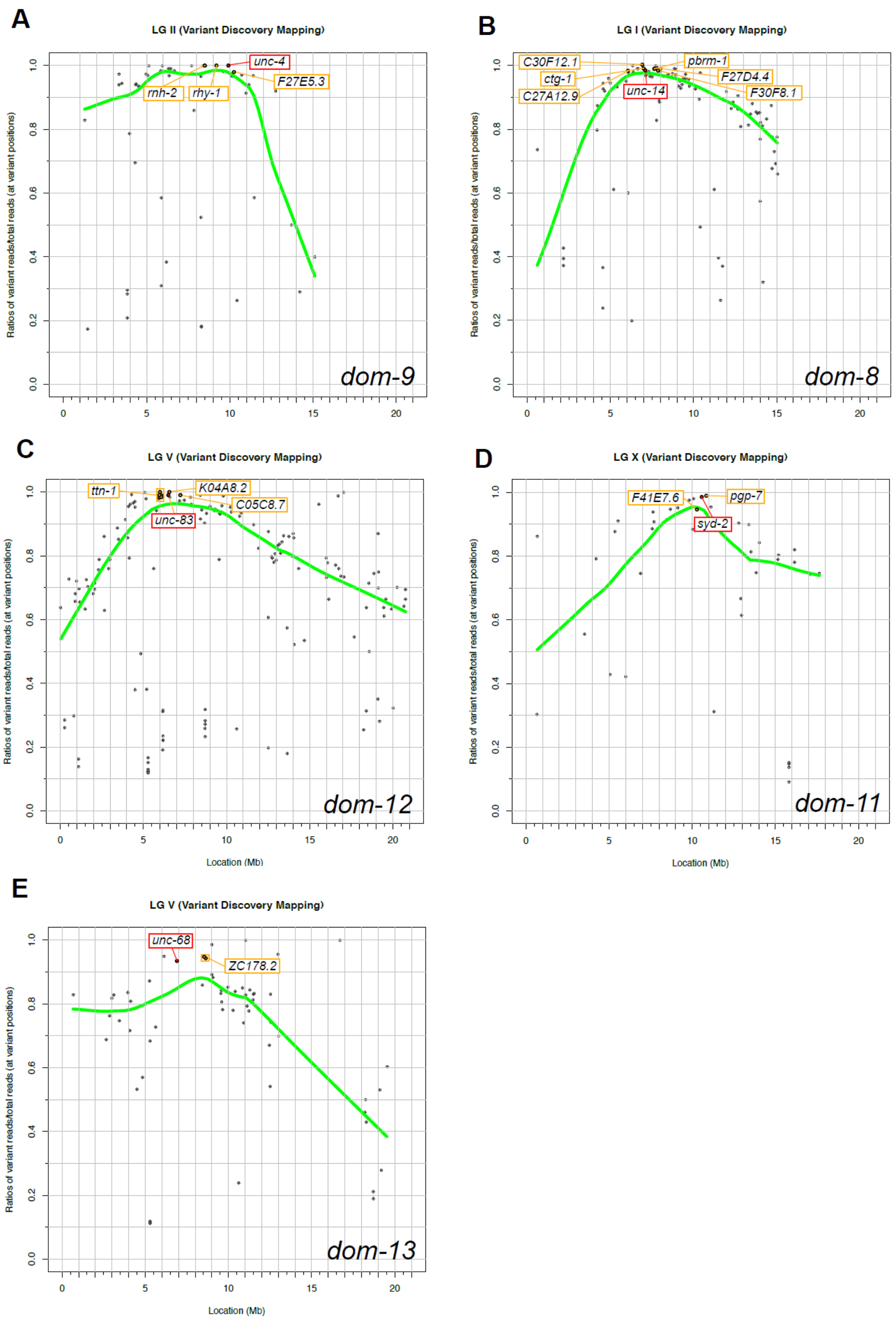

S2 Figure. Variant Discovery Mapping plots

(**A**,**B**,**C**,**D**,**E**) The allele frequencies of high-quality variants (ratio of variants reads/total reads) identified in whole-genome sequenced pools of homozygous mutant recombinants are plotted as a function of genomic coordinates. The green line is a LOESS (locally estimated scatterplot smoothing) local regression to help identify the linked (peak) region. Protein coding mutations located in the peak region are indicated with rectangular labels (red for the causal mutation and yellow for other linked non-causal mutations).
