# Supplementary material for: Identification of avoidance genes through neural pathway-specific forward optogenetics
Source: PLoS Genet. 2019 Dec 31;15(12):e1008509. doi: 10.1371/journal.pgen.1008509 (PMC6938339; doi:10.1371/journal.pgen.1008509)
Supplement: S3 Fig — Reversal analysis results from Figs 3, 4 and 5 pooled in a single heat map. Data are expressed according to the depicted color scale as a fraction of wild type responsiveness. (PDF) [file pgen.1008509.s003.pdf]

S3 Figure

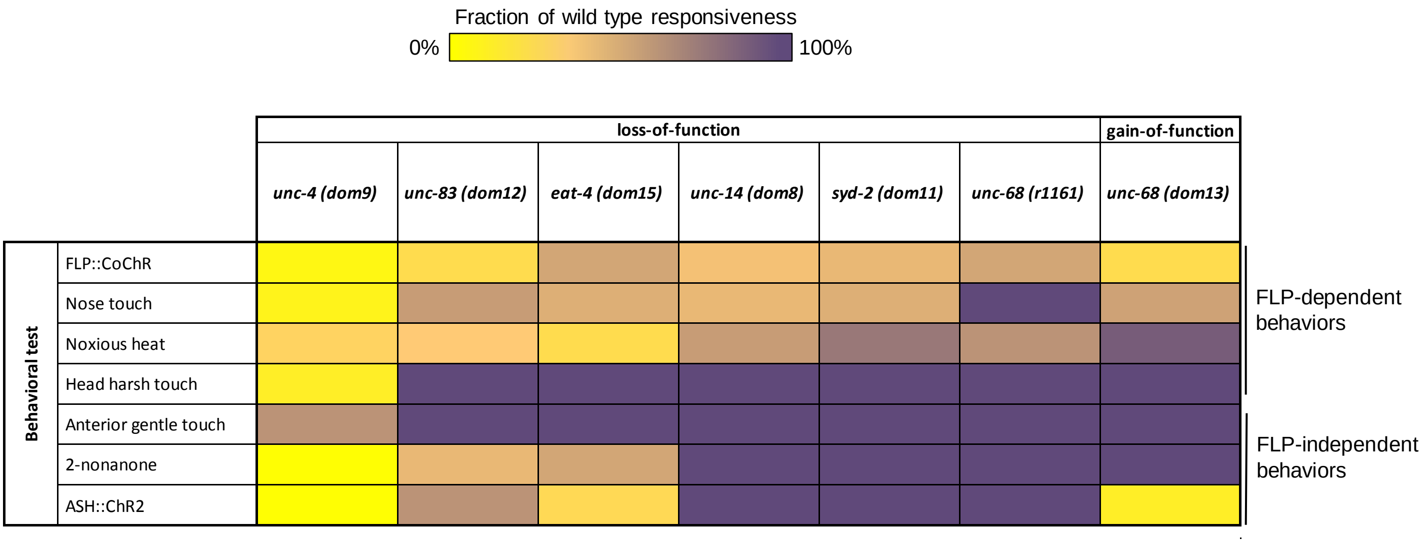

S3 Figure. Overview of behavioral impairments in mutants
Reversal analysis results from Fig. 3, 4 and 5 pooled in a single heat map. Data are expressed according to the depicted color scale as a fraction of wild type responsiveness.
