# Supplementary material for: Identification of avoidance genes through neural pathway-specific forward optogenetics
Source: PLoS Genet. 2019 Dec 31;15(12):e1008509. doi: 10.1371/journal.pgen.1008509 (PMC6938339; doi:10.1371/journal.pgen.1008509)
Supplement: S4 Fig — A: Schematic of the three transgenes used and their roles. B, C: Representative epifluorescence micrographs from a single animal revealing both green and red signals, using a high pass emission filter and a color camera to visualize both channels. Only green signal is detectable in FLP (B, top inset), while red nuclei are detected in PLM (C, lower left inset) and a large number of other neurons (including motor neurons, C, lower right inset). D: Quantification of [rab-3p::wrmScarlet] signal in FLP and motor neurons. FLP nuclei were localized thanks to the mNeonGreen signal. Acquisition for quantification was made with a RFP filter cube to selectively quantify the wrmScarlet signal. Note the interrupted vertical axis. Grey bars indicate averages, with SEM as error bars. *, p < .001 by Student’s t-test. Data are pooled from two independent transgenic lines showing similar results. (PDF) [file pgen.1008509.s004.pdf]

S4 Figure

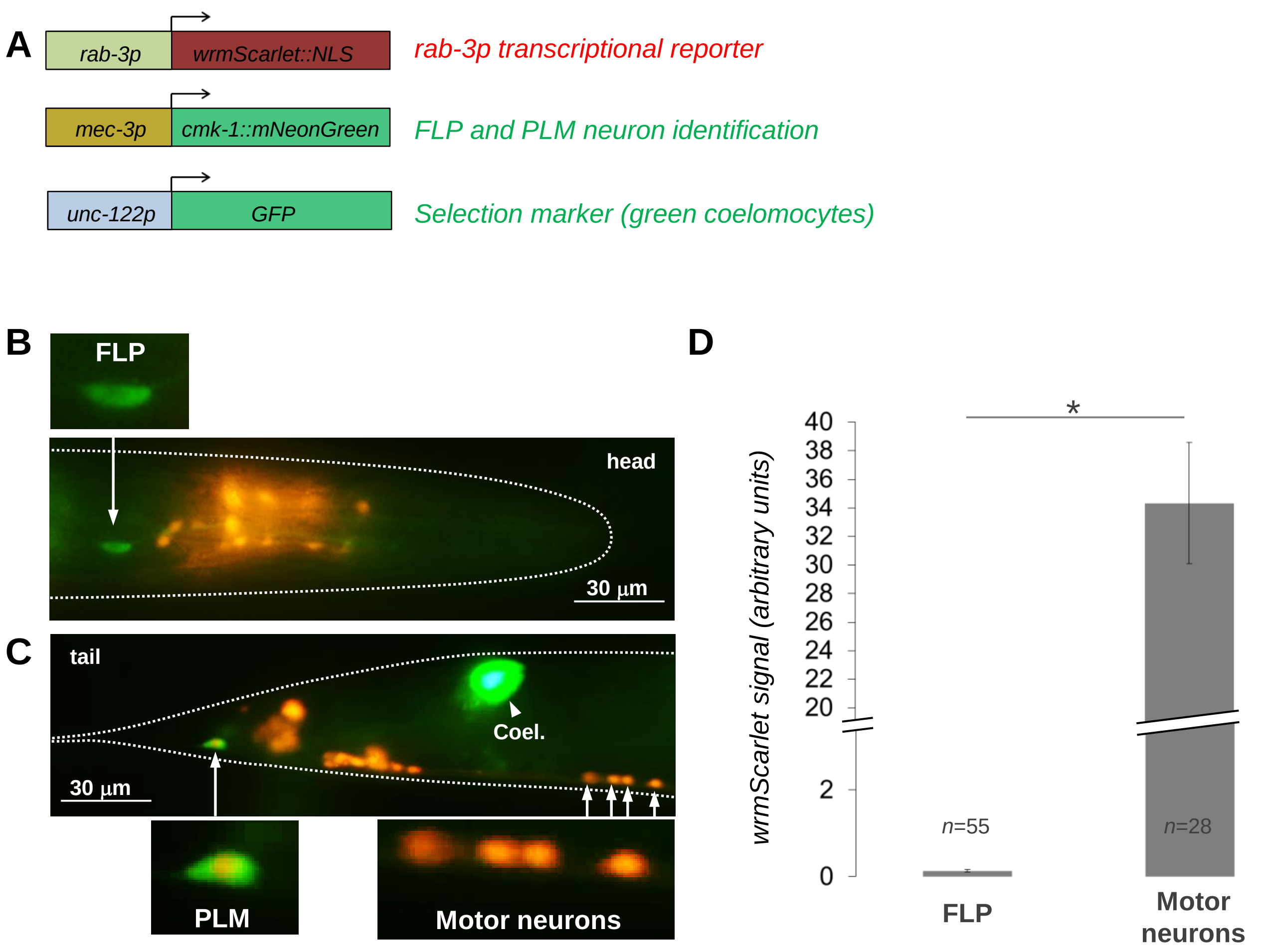

S4 Figure. The *rab-3* promoter used for 'pan-neuronal' *unc-68* rescue drives nearly undetectable expression in FLP
A: Schematic of the three transgenes used and their roles.
B, **C**: Representative epifluorescence micrographs from a single animal revealing both green and red signals, using a high pass emission filter and a color camera to visualize both channels. Only green signal is detectable in FLP (**B**, top inset), while red nuclei are detected in PLM (**C**, lower left inset) and a large number of other neurons (including motor neurons, **C**, lower right inset).
D: Quantification of [rab-3p::wrmScarlet] signal in FLP and motor neurons. FLP nuclei were localized thanks to the mNeonGreen signal. Acquisition for quantification was made with a RFP filter cube to selectively quantify the wrmScarlet signal. Note the interrupted vertical axis. Grey bars indicate averages, with SEM as error bars. *, $p<.001$ by Student's t-test. Data are pooled from two independent transgenic lines showing similar results.
