# Supplementary material for: Identification of avoidance genes through neural pathway-specific forward optogenetics
Source: PLoS Genet. 2019 Dec 31;15(12):e1008509. doi: 10.1371/journal.pgen.1008509 (PMC6938339; doi:10.1371/journal.pgen.1008509)
Supplement: S1 Table — (PDF) [file pgen.1008509.s005.pdf]

S1 Table. New alleles recovered in the genetic screen

Allele	Mutation with flanking genomic sequence
unc-14(dom8)	TTGGACTGCTGGCTGTGCTA(Tto**A**)ATTGTGTTGAAAGAGAAGGT
unc-4(dom9)	AAAATCAAATTTTCAGGTTG(Gto**A**)CAACTGGAAGAGCTCGAATC
syd-2(dom11)	AGTTGGAAAATTATAAGGTG(Cto**T**)AACTGGAAAATGCAGGGCTC
unc-83(dom12)	GTAGCATGAGTGACGGAGAG(Cto**T**)AATTGGGCGTTGTGAGCAGT
unc-68(dom13)	CAAAAATTGAACATTTTTAG(Gto**A**)TTTGATCATCGACGCTTTCG
eat-4(dom15)	GGAAAATGCCGAAAACGTTG(Gto**A**)CTTCTAGCTATTCTTGCAAA
