# Supplementary material for: Identification of avoidance genes through neural pathway-specific forward optogenetics
Source: PLoS Genet. 2019 Dec 31;15(12):e1008509. doi: 10.1371/journal.pgen.1008509 (PMC6938339; doi:10.1371/journal.pgen.1008509)
Supplement: S2 Table — (PDF) [file pgen.1008509.s006.pdf]

S2 Table. Strains used in this study

Mutant and integrated transgenic strains

**Strain name**	**Genotype**	**Comment**
N2	Wild type	Obtained from the CGC
CB120	*unc-4(e120) II*	Obtained from the CGC
MT6308	*eat-4(ky5) III*	Obtained from the CGC
LX748	*osm-9(ky10) ocr-2(ak47) IV*	Obtained from the CGC
CB1472	*mec-6(e1342) I*	Obtained from the CGC
TR2170	*unc-68(r1161) V*	Obtained from the CGC
DAG356	*domIs355[mec-3p::QF, mec-4p::QS, QUAS::CoChR::GFP, unc122p::RFP]*	*[FLP::CoChR]* FLP optogenetic background, used as background for the mutagenesis screen
DAG411	*domIs355; eat-4 (ky5) III*	*eat-4 (ky5)* mutant in FLP optogenetic background
DAG502	*domIs355; unc-14(dom8) I*	New mutant from the screen in FLP optogenetic background, 3x backcrossed with the parental line DAG356
DAG503	*domIs355; unc-4(dom9) II*	New mutant from the screen in FLP optogenetic background, 3x backcrossed with the parental line DAG356
DAG505	*domIs355 ; syd-2(dom11) X*	New mutant from the screen in FLP optogenetic background, 3x backcrossed with the parental line DAG356
DAG506	*domIs355; unc-83(dom12) V*	New mutant from the screen in FLP optogenetic background, 3x backcrossed with the parental line DAG356
DAG515	*domIs355; unc-68(dom13) V*	New mutant from the screen in FLP optogenetic background, 3x backcrossed with the parental line DAG356
DAG517	*domIs355; eat-4(dom15) III*	New mutant from the screen in FLP optogenetic background, 3x backcrossed with the parental line DAG356
DAG618	*domIs355;unc-68(r1161) V*	*unc-68(r1161)* mutant in FLP optogenetic background

DAG658	*domIs355 ; zwIs100[rab-3p::Myc::ryr-1 + myo-3p::GFP];unc-68 (r1161)*	*unc-68(r1161)* mutant in FLP optogenetic background with neuronal rescue transgene
DAG680	*domIs355; zwIs108[myo-3p::Myc::ryr-1 + myo-3p::GFP;unc-68(r1161)*	*unc-68 (r1161)* mutant in FLP optogenetic background with muscle rescue transgene
PHX212	*unc-68 (syb212) V*	*unc-68(syb212)* UNC-68 G4996D mutation made by genome editing (SunyBiotech, China)
DAG476	*domIs355[mec-3p::QF, mec-4p::QS, QUAS::CoCHR, unc122p::RFP]; domSi453[mec-3p::cmk-1::mNeonGreen::3xFlag::unc-54UTR]*	Reporter for *mec-3* expression and FLP morphology evaluation
DAG477	*domIs355[mec-3p::QF, mec-4p::QS, QUAS::CoCHR, unc122p::RFP]; domSi453[mec-3p::cmk-1::mNeonGreen::3xFlag::unc-54UTR];unc-4(dom9)*	Reporter for *mec-3* expression and FLP morphology evaluation
DAG479	*domIs355[mec-3p::QF, mec-4p::QS, QUAS::CoCHR, unc122p::RFP]; domSi453[mec-3p::cmk-1::mNeonGreen::3xFlag::unc-54UTR];unc-68(dom13)*	Reporter for *mec-3* expression and FLP morphology evaluation
DAG485	*domIs355[mec-3p::QF, mec-4p::QS, QUAS::CoCHR, unc122p::RFP]; domSi453[mec-3p::cmk-1::mNeonGreen::3xFlag::unc-54UTR];unc-83(dom12)*	Reporter for *mec-3* expression and FLP morphology evaluation
DAG498	*domIs355[mec-3p::QF, mec-4p::QS, QUAS::CoCHR, unc122p::RFP]; domSi453[mec-3p::cmk-1::mNeonGreen::3xFlag::unc-54UTR];unc-14(dom8)*	Reporter for *mec-3* expression and FLP morphology evaluation
DAG500	*domIs355[mec-3p::QF, mec-4p::QS, QUAS::CoCHR, unc122p::RFP]; domSi453[mec-3p::cmk-1::mNeonGreen::3xFlag::unc-54UTR];syd-2(dom11)*	Reporter for *mec-3* expression and FLP morphology evaluation

DAG483	*domIs355[mec-3p::QF, mec-4p::QS, QUAS::CoCHR, unc122p::RFP]; domSi453[mec-3p::cmk-1::mNeonGreen::3xFlag::unc-54UTR];eat-4(dom15)*	Reporter for *mec-3* expression and FLP morphology evaluation
DAG871	*unc-14(dom8) I*	New mutant from the screen outcrossed to wild type background
DAG872	*unc-4(dom9) II*	New mutant from the screen outcrossed to wild type background
DAG873	*syd-2(dom11) X*	New mutant from the screen outcrossed to wild type background
DAG874	*unc-68(dom13) V*	New mutant from the screen outcrossed to wild type background
DAG875	*eat-4(dom15) III*	New mutant from the screen outcrossed to wild type background
DAG882	*unc-83(dom12) V*	New mutant from the screen outcrossed to wild type background
AQ2235	*lite-1(ce314); ljIs114[Pgpa-13::FLPase,Psra6::FTF::ChR2::YFP] X*	New mutant from the screen in *[ASH:ChR2]* optogenetic background
DAG814	*lite-1(ce314); ljIs114[Pgpa-13::FLPase, Psra6::FTF::ChR2::YFP] X; unc-68(r1161) V*	New mutant from the screen in *[ASH:ChR2]* optogenetic background
DAG919	*lite-1(ce314); ljIs114[Pgpa-13::FLPase, Psra-6::FTF::ChR2::YFP] X ; eat-4(ky5)III*	New mutant from the screen in *[ASH:ChR2]* optogenetic background
DAG920	*lite-1(ce314); ljIs114[Pgpa-13::FLPase, Psra-6::FTF::ChR2::YFP] X; unc-68(dom13) V*	New mutant from the screen in *[ASH:ChR2]* optogenetic background
DAG921	*lite-1(ce314); ljIs114[Pgpa-13::FLPase, Psra-6::FTF::ChR2::YFP] X; unc-4(dom9) II*	New mutant from the screen in *[ASH:ChR2]* optogenetic background
DAG922	*lite-1(ce314); ljIs114[Pgpa-13::FLPase, Psra-6::FTF::ChR2::YFP] X; unc-83(dom12) V*	New mutant from the screen in *[ASH:ChR2]* optogenetic background
DAG923	*lite-1(ce314); ljIs114[Pgpa-13::FLPase, Psra-6::FTF::ChR2::YFP] X; unc-14(dom8) I*	New mutant from the screen in *[ASH:ChR2]* optogenetic background
DAG970	*lite-1(ce314); ljIs114[Pgpa-13::FLPase, Psra-6::FTF::ChR2::YFP] syd-2(dom11) X*	New mutant from the screen in *[ASH:ChR2]* optogenetic background

Strains carrying non-integrated extra-chromosomal arrays

Strain	Genotype	Comment
DAG624-625	*domIs355; unc-14 (dom8) I; domEx624-625[unc-14p::unc 14CDS::SL2::mCherry, unc122p::GFP]*	*unc-14(dom8)* rescue with the endogenous *unc-14* promoter driving *unc-14* coding sequence (CDS)
DAG609	*domIs355; unc-4(dom9) II; domEx609[unc-4p::unc-4CDS::SL2::mCherry::unc122p::GFP]*	*unc-4(dom9)* rescue with the endogenous *unc-4* promoter driving *unc-4* coding sequence (CDS)
DAG629-630	*domIs355; syd-2(dom11) X; domEx629-630[syd-2p::syd2CDS::SL2::mCherry, unc122p::GFP]*	*syd-2(dom11)* rescue with the endogenous *syd-2* promoter driving *syd-2* coding sequence (CDS)
DAG631-632	*domIs355; unc-83(dom12) V;domEx631-632[W01A11cosmid, unc122p::GFP]*	*unc-83(dom12)* rescue with *unc-83* cosmid W01A11
DAG638/688	*domIs355; unc-68(dom13) V; domEx638/688[WRM069cA02fosmid, unc122p::GFP]*	*unc-68(dom13)* rescue with *unc-68* fosmid WRM069cA02
DAG952	*domIs355; eat-4(dom15) III; domEx952(F23K23 fosmid, unc122p::GFP)*	*eat-4(dom15)* rescue with *eat-4* fosmid F23K23
DAG787-788/861	*domIs355; unc68(r1161) V; domEx787-788/861[unc-68p_QF,SL2mCherry_ unc122p::GFP];QUAS_unc-68fosmid(exon1(fs))]*	*unc-68(r1161)* rescue with frameshift *unc-68* fosmid driven by *unc-68* promoter
DAG869	*domIs355; unc-68(r1161) V; domEx869[mec-3p::QF::SL2::mCherry, unc122p::GFP]; domEx681[QUAS_unc-68fosmid]*	*unc-68(r1161)* rescue with *unc-68* fosmid driven by *mec-3* promoter
DAG683-684	*domIs355 ; unc-68(r1161) V; domEx683-684[*WRM069cA02*fosmid_ unc122p::GFP]*	*unc-68 (r1161)* rescue with fosmid WRM069cA02
DAG778-779	*domIs355; domEx778-779[unc-68p_unc-68 RNAisense,SL2mCherry_ unc122p::GFP]*	*unc-68* RNA*i* sense control with *unc-68* promoter
DAG780-781	*domIs355; domEx778-779[mec-3p_unc-68 RNAisense,SL2mCherry_ unc122p::GFP]*	*unc-68* RNA*i* sense control with *mec-3* promoter
DAG832-834	*domIs355]; domEx832-834[mec-3p::unc-68RNAi_sense::SL2mCherry, mec-3p::unc-68RNAi_antisense::SL2mCherry, unc122p::GFP]*	*unc-68* RNA*i* double-strand with *mec-3* promoter

DAG837-839	*domIs355; domEx837-839[unc-68p::unc-68RNAi_sense::SL2mCherry, unc-68p::unc-68RNAi_antisense::SL2mCherry, unc122p::GFP]*	*unc-68* RNA*i* double-strand with *unc-68* promoter
AQ2145	*ljEx19[pegl-46::YC2.3 lin-15(+)]*	Calcium response assessment
DAG815	*ljEx19[pegl-46::YC2.3 lin-15(+)]; unc-68(dom13) V*	Calcium response assessment
DAG747	*ljEx19[pegl-46::YC2.3 lin-15(+)]; unc-68(r1161) V*	Calcium response assessment
DAG960-961	*domSi439[mec3p::cmk1::mNeonGreen::3xFlag::unc-4UTR] II; domEx960961[rab-3_1208prom:: NLSegl-13::wrmScarlet::unc-54UTR]*	*rab-3p* expression reporter in red with FLP identification in green
DAG1071-1072	*domIs355; unc-14 (dom8) I; domEx1071-1072[mec-3p::unc 14CDS::SL2::mCherry, unc122p::GFP]*	*unc-14(dom8) rescue with mec-3 promoter driving unc-14 coding sequence (CDS)*
DAG1082-1083	*domIs355; unc-4(dom9) II; domEx1082-1083[mec-3p::unc-4CDS::SL2::mCherry::unc122p::GFP]*	*unc-4(dom9) rescue with mec-3 promoter driving unc-4 coding sequence (CDS)*
DAG1070; DAG1073-1074	*domIs355; syd-2(dom11) X; domEx1070;1073-1074[mec-3p::syd-2CDS::SL2::mCherry, unc122p::GFP]*	*syd-2(dom11) rescue with mec-3 promoter driving syd-2 coding sequence (CDS)*
DAG1076-1078	*domIs355; eat-4(dom15) III; domEx1076-1078 [eat-4-p::eat-4CDS::SL2::mCherry, unc122p::GFP]*	*eat-4(dom15) rescue with the endogenous eat-4 promoter driving eat-4 coding sequence (CDS)*
DAG1079-1081	*domIs355; eat-4(dom15) III; domEx1079-1081[mec-3p::eat-4CDS::SL2::mCherry, unc122p::GFP]*	*eat-4(dom15) rescue with mec-3 promoter driving eat-4 coding sequence (CDS)*
